# Radiogenomic classification for MGMT promoter methylation status using multi-omics fused feature space for least invasive diagnosis through mpMRI scans

**DOI:** 10.1038/s41598-023-30309-4

**Published:** 2023-02-25

**Authors:** Shahzad Ahmad Qureshi, Lal Hussain, Usama Ibrar, Eatedal Alabdulkreem, Mohamed K. Nour, Mohammed S. Alqahtani, Faisal Mohammed Nafie, Abdullah Mohamed, Gouse Pasha Mohammed, Tim Q. Duong

**Affiliations:** 1 Department of Computer and Information Sciences, Pakistan Institute of Engineering and Applied Sciences, Islamabad, Pakistan; 2 Department of Computer Science and IT, Neelum Campus, The University of Azad Jammu and Kashmir, Muzaffarabad, Azad Kashmir Pakistan; 3 Department of Computer Science and IT, King Abdullah Campus, The University of Azad Jammu and Kashmir, Muzaffarabad, Azad Kashmir Pakistan; 4 Department of Radiology, Albert Einstein College of Medicine and Montefiore Medical Center, 111 East 210th Street, Bronx, NY 10467 USA; 5 Farooq Hospital, Lahore, Pakistan; 6 Department of Computer Sciences, College of Computer and Information Sciences, Princess Nourah Bint Abdulrahman University, P.O. Box 84428, Riyadh, 11671 Saudi Arabia; 7 Department of Computer Sciences, College of Computing and Information System, Umm Al-Qura University, Mecca, Saudi Arabia; 8 Radiological Sciences Department, College of Applied Medical Sciences, King Khalid University, Abha, 61421 Saudi Arabia; 9 Department of Computer Science, College of Science and Humanities at Alghat, Majmaah University, Al-Majmaah, 11952 Saudi Arabia; 10 Research Centre, Future University in Egypt, New Cairo, 11845 Egypt; 11 Department of Computer and Self Development, Preparatory Year Deanship, Prince Sattam Bin Abdulaziz University, AlKharj, Saudi Arabia

**Keywords:** Cancer, Diseases, Medical research, Engineering

## Abstract

Accurate radiogenomic classification of brain tumors is important to improve the standard of diagnosis, prognosis, and treatment planning for patients with glioblastoma. In this study, we propose a novel two-stage MGMT Promoter Methylation Prediction (MGMT-PMP) system that extracts latent features fused with radiomic features predicting the genetic subtype of glioblastoma. A novel fine-tuned deep learning architecture, namely Deep Learning Radiomic Feature Extraction (DLRFE) module, is proposed for latent feature extraction that fuses the quantitative knowledge to the spatial distribution and the size of tumorous structure through radiomic features: (GLCM, HOG, and LBP). The application of the novice rejection algorithm has been found significantly effective in selecting and isolating the negative training instances out of the original dataset. The fused feature vectors are then used for training and testing by *k*-NN and SVM classifiers. The 2021 RSNA Brain Tumor challenge dataset (BraTS-2021) consists of four structural mpMRIs, viz. fluid-attenuated inversion-recovery, T1-weighted, T1-weighted contrast enhancement, and T2-weighted. We evaluated the classification performance, for the very first time in published form, in terms of measures like accuracy, F_1_-score, and Matthews correlation coefficient. The Jackknife tenfold cross-validation was used for training and testing BraTS-2021 dataset validation. The highest classification performance is (96.84 ± 0.09)%, (96.08 ± 0.10)%, and (97.44 ± 0.14)% as accuracy, sensitivity, and specificity respectively to detect MGMT methylation status for patients suffering from glioblastoma. Deep learning feature extraction with radiogenomic features, fusing imaging phenotypes and molecular structure, using rejection algorithm has been found to perform outclass capable of detecting MGMT methylation status of glioblastoma patients. The approach relates the genomic variation with radiomic features forming a bridge between two areas of research that may prove useful for clinical treatment planning leading to better outcomes.

## Introduction

The division of brain cells, in billions, is declared tumorous in case it is uncontrollable forming abnormal regions, leading to the cancers’ highest mortality rates worldwide for adults as well as children^1^. The tumor localization in the brain with its growth rate is a highly unpredictable entity and is broadly classified as primary and secondary tumors. The former, with its origin inside the brain, is the deadliest one, and malignant most of the time. The gliomas cover 80% of the primary brain tumors (in Grades I to IV only Grade I grows slowly, and is benign)^2,3^. The gliomas, Grades II and III grow quickly and frequently require prompt treatment. Grade-IV gliomas, the most aggressive type, also known as glioblastoma (GB), are the most challenging for prognosis and better clinical outcomes. In adults, GB and diffuse astrocytic glioma, due to extreme intrinsic heterogeneity in shape and microscopic anatomy, are the most dangerous tumors of the central nervous system. It has been observed that the GB patients’ prognosis, irrespective of the use of numerous treatment options, has had no substantial improvement during the last 20 years^4–10^. The World Health Organization (WHO) released details about the central nervous system tumors classification, and emphasized the integrated diagnostics utility, highlighting the clinical tumor diagnosis as integrating molecular-cytogenetic features^11^. O^6^-methylguanine-DNA methyltransferase (MGMT) is an enzyme for DNA repair that plays a vital role in chemoresistance to alkylating agents and is a promising prognostic factor predicting chemotherapy response based on the methylation of the promoter for an early diagnosed GB^12–14^. In this respect, the most common courses of treatment for GB include surgery, radiotherapy, and adjuvant chemotherapy^15^. The effectiveness of chemotherapy is often tied to the promotor methylation status of MGMT, an important biomarker, which functions as a repairing mechanism for guanine nucleotides and prevents cell death caused by alkylating agents^12,16^. In an average scenario, the presence of the MGMT enzyme is beneficial since it prevents DNA damage. In GB patients, however, the presence of MGMT decreases the effectiveness of chemotherapy by rendering the alkylating chemotherapeutic agents ineffective. This work is related to the prediction of the MGMT promoter methylation status ("Preprocessing") causing MGMT gene silencing, where its presence leads to favorable results in GB patients being treated with alkylating agent chemotherapy by stopping cell division through cross-linking DNA strands. Therefore, predicting the methylation status of MGMT promoters in GB can support further decision-making and treatment plan, and it is the sole objective of this research endeavor.

Presently, the minor structural details that are challenging to discriminate by computed tomography (CT) are detected by another non-invasive technique, namely magnetic resonance imaging (MRI). In the case of complicated GB MRI scans, the manual analysis by expert radiologists and physicians is tedious and time taking^17^. The complex cases need to compare the tumorous region with neighboring regions which leads to improving the perceptual information stored in the image for improved classification. This situation is impracticable in the case of a large number of images being dealt with using manual techniques. Early GB detection with reliable prediction results is important for the health of a subject^11^. Consequently, novel approaches are always the main source of attraction for cohorts working critically for prompt and reliable tumor detection. Machine learning (ML) and one of its variants, namely deep learning (DL), is the key enabler of artificial intelligence (AI) for discriminative feature extraction turning the images into useful information.

The RSNA ASNR MICCAI Brain Tumor Segmentation BraTS 2021 challenge (BraTS-2021 dataset) is based on multi-institutional multi-parametric Magnetic Resonance Imaging (mpMRI) scans^18^. This article is related to the prediction of MGMT promoter methylation status, a genetic characteristic of glioblastoma, using baseline MRI scans done before and while preparing for a surgical operation. In our work, we propose a novel classification framework distinguishing either MGMT methylated or MGMT unmethylated tumors using a challenging BraTS-2021 dataset. The former class is designated as (1, or MGMT +) while the latter is categorized as (0, or MGMT−).

The current practice for genetic analysis of cancer tissue samples is to use surgery. Further, the tumor genetic characterization requires weeks before a conclusion is reached^18^. The consequence of the results may lead to subsequent surgery. The notion here is to predict the cancer genetics using magnetic resonance imaging, namely radiogenomics, that might improve the therapy results along with the reduction of potential surgical treatments. The success of radiogenomics would lead to alleviating brain cancer miseries by least invasive measures for the respective diagnoses and treatments. This new treatment strategy, before any surgery, seems to have the potential to improve the prospects of management and survival of brain cancer patients.

The MGMT gene is regulated by an epigenetic mechanism: the methylation of the CpG island of the MGMT promoter. The role of MGMT promoter methylation is to suppress the MGMT genes’ expression thereby decreasing MGMT enzyme function in the cell. Due to the ability of MGMT methylation to increase the effectiveness of chemotherapy, the status of MGMT methylation is often taken into consideration when determining the course of treatment^18–20^. The traditional method to determine MGMT methylation status involves the extraction of tumor tissue through surgery. After extraction, the determination of the methylation status of the tumor is a time (up to weeks) taking process. Further, after determination, additional invasive procedures may be necessary to discern the optimal treatment method^18,21^.

Radiogenomic diagnosis of MGMT methylation testing aids in decreasing the invasiveness of current testing procedures. Radiogenomics aims to predict cellular genomics with the use of a tissue’s phenotypic image characteristics^22,23^. In gliomas, radiomics is commonly used to predict survival, and evaluate the potential of chemotherapeutic treatments in treatment^24^. Several radiogenomic models have been developed to predict MGMT methylation status in GB patients^25–27^. However, radiogenomic models are susceptible to a lack of standardization because of the variation between methodology, software, and radiologists’ readings^23^. Recently, Zhang et al.^28^ introduced a data-sharing scheme based on blockchain with fine-grained access control. They separated the public and private parts of electronic medical records, which are subsequently encrypted separately by symmetric searchable encryption (SSE). The symmetric keys used in SSE technology were encrypted by attribute-based encryption. This helped patients to share data without any risk. For GB, the radiogenomic approach to MGMT methylation testing consists of evaluating magnetic resonance images (MRIs) of the brain^29^. To expand upon current radiogenomic analysis methods, artificial intelligence can be employed to construct complex predictive deep learning radiogenomic models^30^.

Deep convolutional neural networks are employed for a vast array of tasks, including medical image analysis^31,32^ and image classification^14,33^. Deep learning architecture has encoding blocks ordered in multiple layers. The feature maps in lower layers are forwarded to subsequent layers with increased complexity order. A convolutional neural network (CNN)^34^ massively reduces the number of neurons due to sparse interaction in comparison to shallow neural networks. The transfer learning methodology based on CNN is well proven for quite some time^35–39^ and has been extensively used in the analysis of different imaging databases^37,38,40,41^, neuroimaging^42^, MRI, CT (Computed Tomography)^36^, and ultrasound images^43^. Transfer learning using CNN based on AlexNet and GoogleNet for the ImageNet dataset is well known deep learning approach^44^. The CNNs are extensively used in vision-related applications including object detection^45^, spanning classification^46^, and segmentation^47^. The combination of data pre-processing and augmentation with transfer learning can be helpful for improved classification results. In our case, since the dataset is enormous, only pre-processing is seeming of great value along with a fine-tuned CNN architecture.

The features used as the source of training essence should have discrimination ability that would be exploited to predict the regions across the hyperplane with a maximum confidence level as the target label. In this context, there is a gap for efficient and robust automated systems for brain tumor detection using MRI. Hsieh et al.^48^ defined brain tumor categories based on region-of-interest (ROI), feature selection, and feature extraction. They used local textural features including global histogram moments using 107 images of gliomas (73 low-grade; 34 high-grade). The work, however, was reported on a limited dataset and lacking features based on other static feature extraction methods. Cheng et al.^49^ used a T1-weighted Contrast-Enhanced brain MRI dataset^50^ having three types of tumors (glioma, pituitary, and meningioma), experimenting with three feature extraction methods, namely intensity histogram, bag-of-words (BoW), and gray level co-occurrence matrix (GLCM), finding that BoW performs relatively better at a higher computational cost. The accuracy was limited due to the absence of preprocessing scenarios that could lead to improved discriminative features. The hybrid of solution spaces for the three characteristic feature sets was not explored. Similarly, Sachdeva et al.^51^ extracted color and textural features based on segmented ROIs, using the genetic algorithm for features’ selection with optimum fitness level, and reported the accuracy as 94.90% using a genetic algorithm-based artificial neural network (GA-ANN). However, for large datasets, the colored images have different color tints necessitating the use of staining procedures as an essential step. Further, the dynamic features need to be explored with deep learning algorithms to address enhanced discrimination features.

Claro et al.^52^ used hybrid feature space formed by textural features, like Tamura (coarseness, contrast, directionality, line-likeness, regularity, and roughness), gray level run length matrix (GLRLM), histogram of oriented gradients (HOG), morphology, local binary patterns (LBP), merging the extracted features using seven CNN architectures for glaucoma classification. Their feature space was based on 30,862 dimensions, which was squeezed by the gain ratio for arranging the features according to their performance concluding in an optimum setting for glaucoma detection. They found the GLCM descriptor with transfer learning-based features to be the most effective for their specific problem structure. The work needs to be explored on multi-parametric and multi-institutional datasets, for larger and more diverse datasets, with dynamic features using residual feature maps concatenated in the successive layers. Garcia et al.^53^ solved the problem of imbalanced datasets by using ensemble classifiers with feature space partitioning. The parameters of the partitioning were optimized by using a hybrid metaheuristic method, called GACE which combined a genetic algorithm (GA) with a cross-entropy (CE) method. More elaborative work using generative adversarial networks (GAN) can be used to tackle the underlying problem of class imbalance. Shaban et al.^54^ introduced a hybrid feature selection methodology that extracts the features with optimum characteristics using COVID-19 CT images. The feature selection is based on fast and accurate selection stages. They used an enhanced version of *k*-NN that is not trapped due to solid heuristics in choosing the neighbors of the tested subject. The work can be explored using other ML classifiers, along with DL classification techniques to involve the features based on high-level abstraction layers.

In this research, we propose a novel two-stage MGMT Promoter Methylation Prediction (MGMT-PMP) system, that precisely quantifies the image structure of GB in patients from the evaluation of FLAIR, T1w, T2, and T1Gd mpMRIs. We have selected the popular feature types, viz. GLCM, HOG, and LBP, and fused these features with novice deep learning features forming a hybrid feature set (HFS) differing vis-à-vis in three aspects. Firstly, it engages a novel Deep Learning Radiomic Feature Extraction (DLRFE) module that extracts dynamic features based on the problem structure into the classification process leading to promising results. Secondly, it provides different categories of second-order statistics and local textural features exploiting the positive aspects of each category of feature extraction modules. Third, the system is based on filtering that uses the rejection algorithm for removing redundant and irrelevant features from the RSNA dataset thereby improving the discrimination or variance and leading to its quick convergence. A comparison with recent techniques is also presented for performance analysis.

The key contributions of this research work are summarized as follows:

- This work is related to the MGMT promotor methylation status affecting the efficiency of chemotherapy in GB patients where ‘MGMT+’ status increases the effectiveness of chemotherapy.
- A novel two-stage prediction system for MGMT promoter methylation status, that precisely quantifies the image structure of glioblastoma in patients using FLAIR, T1w, T2, and T1Gd mpMRIs.
- A novel deep learning-based feature extraction module that extracts dynamic features based on the problem structure.
- The hybrid feature set formation by fusing deep features with static features of the origin GLCM, HOG, and LBP.
- RSNA ASNR MICCAI Brain Tumor Segmentation BraTS 2021 challenge was used for radiogenomic classification with 348,642 mpMRI scans for the very first time in published form (using performance measures, viz. accuracy, F_1_-score, and Matthews correlation coefficient).
- The rejection algorithm is introduced for removing redundant and irrelevant features from the dataset.
- A detailed complexity analysis of the individual and combinatorial hybrids formed by the fusion of dynamic and static feature sets.
- A comparison of the proposed work with other state-of-the-art techniques.

The paper organization follows; "Importance in the Clinical management and the survival of GB patients" briefly details the impact of the study on the survival of GB patients with clinical management, "Material and methods" is dedicated to materials and methods, "Results and discussion" details the results and discussion, followed by Conclusions in section "Conclusions". The abbreviations used throughout this article are illustrated in Table 1.

**Table 1:** Abbreviations with acronyms used in the text.

Acronym	Abbreviations
BraTS	RSNA ASNR MICCAI brain tumor segmentation
BS	Block size for HOG feature extraction
CaPTk	Cancer imaging phenomics toolkit
CS	Cell size for HOG feature extraction
CEL	Cross entropy loss
CTP	Clinical trials processor
DLFET	Deep learning feature extraction time
DICOM	Digital imaging and communications in medicine
FeTS	Federated tumor segmentation
GA-ANN	Genetic algorithm-artificial neural network
GB	Glioblastoma tumor
GLCM	Grey level co-occurrence matrix
HOG	Histogram of oriented gradients
HFS	Hybrid feature set
*k*-NN	*k*-nearest neighbors
LBP	Local binary patterns
MCC	Mathews correlation coefficient
mpMRI	*Multi-parametric* magnetic resonance imaging
MGMT	O^6^-methylguanine-DNA methyltransferase
MLTrgT	Machine learning training time
MLTstT	Machine learning testing time
NIfTI	Neuroimaging informatics technology initiative
PHI	Protected health information
PMP	Promoter methylation prediction
RA	Rejection algorithm
RBF	Radial basal function
RCM	Radiotherapy and chemotherapy map
RSNA	Radiological society of North America
SNE	Stochastic neighborhood embedding
SVM	Support vector machine
T1w	T1 weighted
T1wCE	T1 weighted contrast enhanced
T2W	T2 weighted

## Importance in the clinical management and the survival of GB patients

Gliomas with varying levels of heinous symptoms have been declared a serious threat to the central nervous system. The BraTS-2021 focuses on the molecular representation and structure of the underlying tumor in intraoperative neurosurgery and serves preoperative ground using mpMRI data^18,40^. Its objective has been the localization of the brain tumor sub-regions that are microscopically distinct in structure. The identification of tumor boundaries in MRI is of importance in surgical treatment planning, intraoperative brain incision to monitor the tumor growth, and planning the radiotherapy and chemotherapy maps (RCM) following the surgical treatments. Irrespective of the poor prognosis of GB patients, the tumor’s MGMT promoter methylation status, which can be found by radiogenomic classification of mpMRI scans, is extremely important to indicate the chemotherapy response prediction. Further, due to the proposed fine-tuned framework, using low-cost GPU-based portable machines would greatly help medical practitioners assist consumers along with the support system to manage post-surgical treatment more effectively.

Some recent cohorts have focused on the information freshness notion. Yang et al.^55^ offset the COVID-19 effects by controlling the diffusion of the epidemic by introducing Age of Information (AoI), a measure for the quantification of information freshness, an optimization scheme using artificial intelligence-based diagnostic bots. Initially, they formed a health state monitoring system where the diagnostic biosensing data was transmitted through bots using edge servers. It was followed by the derivation of AoI problem in a closed form. They also proposed algorithms for bot placement and channel selection using stochastic learning.

## Material and methods

**Figure 1:**
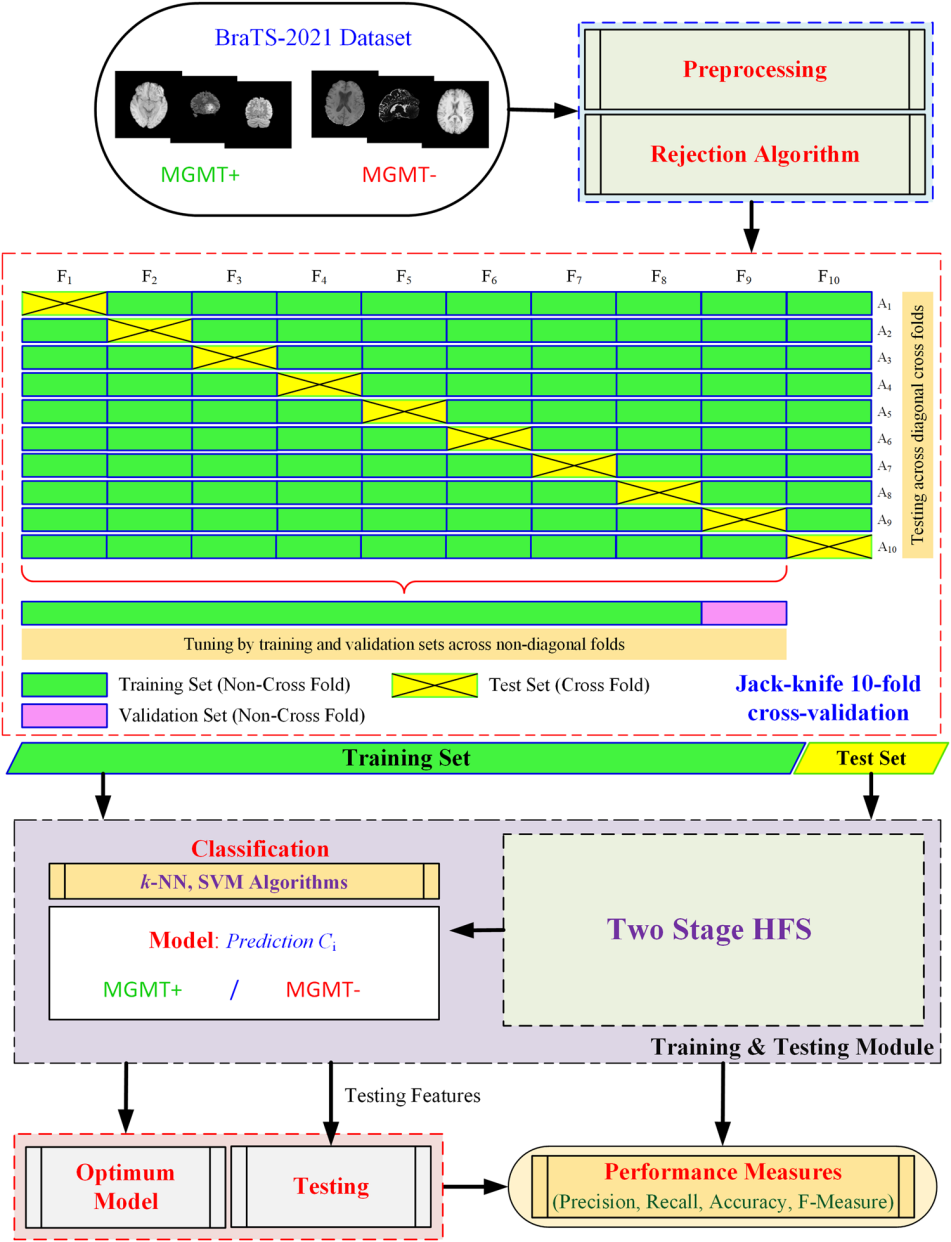
The proposed MGMT promoter methylation prediction (MGMT-PMP) system based on DLRFE forming a two-stage HFS.

The proposed framework (Fig. 1), namely MGMT Promoter Methylation Prediction (MGMT-PMP) System, forms a highly discriminative feature set, HFS, in two stages. In stage one, the notion is to extract features using the DLRFE module where the features from the last flattened layers of the convolutional neural network are acquired, whereas, in stage two the second-order statistics and local textural features are extracted. The features from these stages are merged to form HFS. The feature fusion is further used for radiogenomic classification by a strong ML classifier, like SVM or *k*-NN algorithms. The dataset details, preprocessing, and feature extraction finally follow the prediction model in this section.

### Dataset

**Figure 2:**
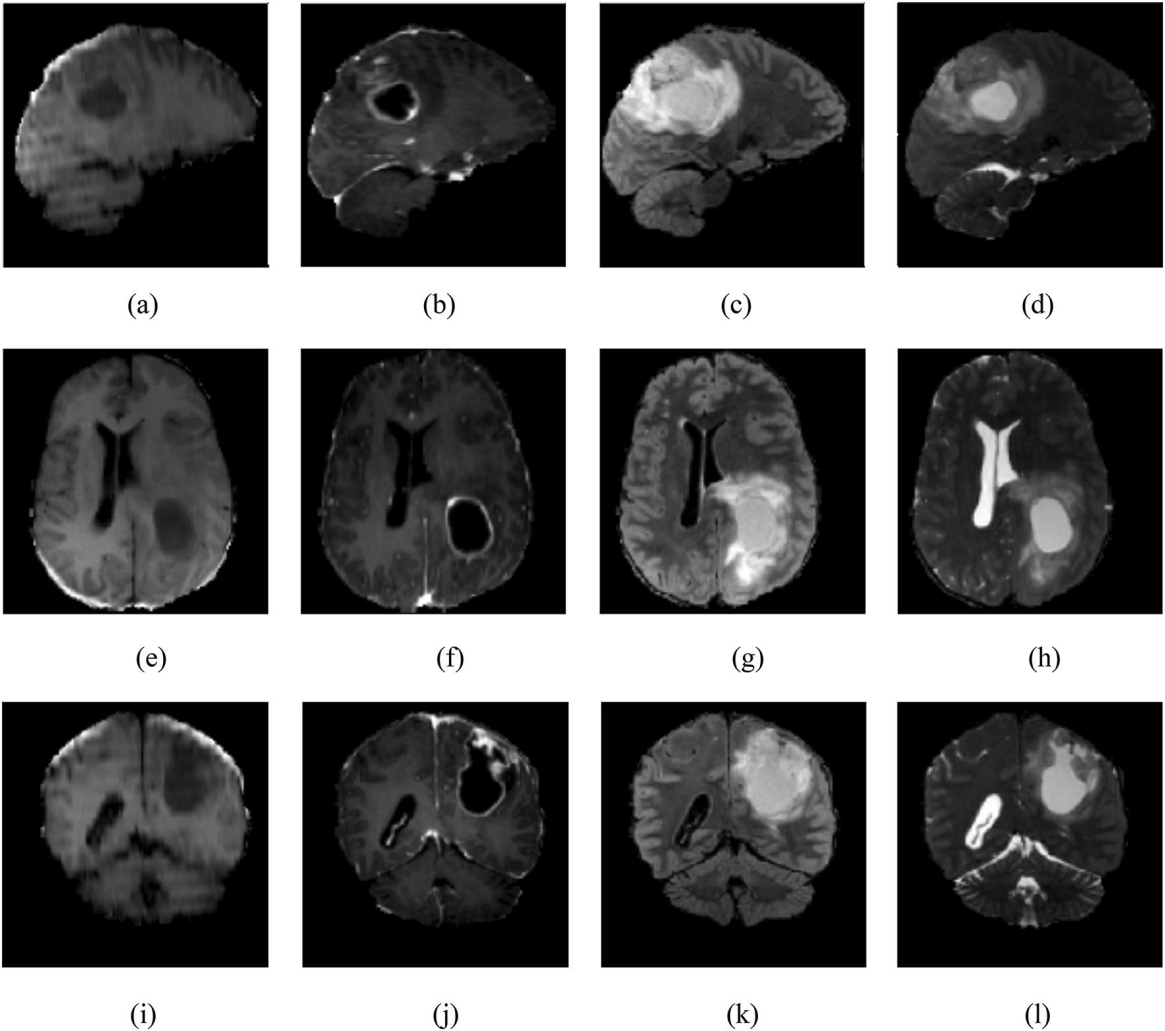
BraTS-2021 mpMRI scans from top to bottom: sagittal, axial and coronal view: first, second and third columns show (**a**,**e**,**i**) T1w, (**b**,**f**,**j**) T1wCE, (**c**,**g**,**k**) FLAIR, and (**d**,**h**,**l**) T2w images respectively.

The proposed framework was analyzed on a publicly available RSNA-MICCAI Brain Tumor Radiogenomic Classification dataset (BRATS-2021)^56^, for a featured code competition that was introduced for the treatment of brain cancer to predict the state of the genetic biomarker. The BraTS-2021 is going to be a common benchmark for GB segmentation algorithms using multi-institutional and multi-parametric Magnetic Resonance Imaging (mpMRI) images of 2000 patients suffering from glioma. The preoperative MRI images were divided into training, validation, and testing cohorts. For the classification task, the target labels, based on the MGMT promoter methylation status, are provided for 585 subjects. The testing cohort is not accessible currently and the validation data for 87 subjects are provided without labels. We have used the training data provided with labels and further partitioned it for testing by cross-validating the results ten folds. The available modalities are T1 weighted (T1w), T1 weighted contrast enhancement (T1wCE), T2 weighted (T2w), and fluid-attenuated inversion-recovery (FLAIR). The two tasks that BraTS-2021 focuses on are: first, segmentation of tumor sub-regions, and second, radiogenomic classification of the MGMT promoter methylation status^18^. Some rescaled mpMRI images from the BraTS-2021 dataset, as illustrated in Fig. 2, showing from top to bottom: sagittal, axial, and coronal views, and the variation in columns represents the intra-class variance of the dataset. The specifications for training and testing instances in BRATS-2021, from the given “train” directory, have been illustrated in Table 2. Similar information files leading to redundant data are removed using the rejection algorithm (RA). The RA trimming effect on instances has been illustrated in the lower part of Table 2 where the specifications of the reduced dataset have been shown ("Preprocessing").

**Table 2:** Statistics of RSNA-MICCAI Brain Tumor Radiogenomic Classification dataset (with and without RA).

Dataset	Instances	Classes	Total instances (class wise)	Rejected instances (class wise)	Training* instances (80%)	Testing** instances (20%)	Significance (MGMT promoter methylation)
BRATS-2021 dataset (original)^18^	348,642	0 (MGMT−)	152,852	×	122,282	30,570	Not present
		1 (MGMT +)	195,790	×	156,632	39,158	Present
Improved BRATS-2021 dataset (with RA)	253,891	0 (MGMT−)	111,960	40,892	89,568	22,392	Not present
		1 (MGMT +)	141,931	53,859	113,545	28,386	Present

*Total Training Instances (Original dataset): 278,914.

Total Training Instances (Improved dataset): 203,113.

**Total Testing Instances (Original dataset): 69,728.

Total Testing Instances (Improved dataset): 50,778.

In the absence of a testing cohort, the BraTS-2021 dataset is constituted of 348,642 training instances. The sharing of this dataset can be useful provided that its security-related issues are addressed The large amount of data generated by heterogeneous Internet of Medical Things (IoMT) may be dispatched to the cloud servers for pathological analysis and diagnosis. This direct mode is convenient for both patients and clinicians, however, the open communication channel has numerous security and privacy issues. In this context, recently Wang et al.^57^ perceived the physical-layer security and over-centralized server problem in wireless medical sensor networks, and proposed a reliable authentication protocol using cuttingedge blockchain technology and physically unclonable functions. Further, the biometric information was dealt with a fuzzy extractor scheme.

### Preprocessing

**Figure 3:**
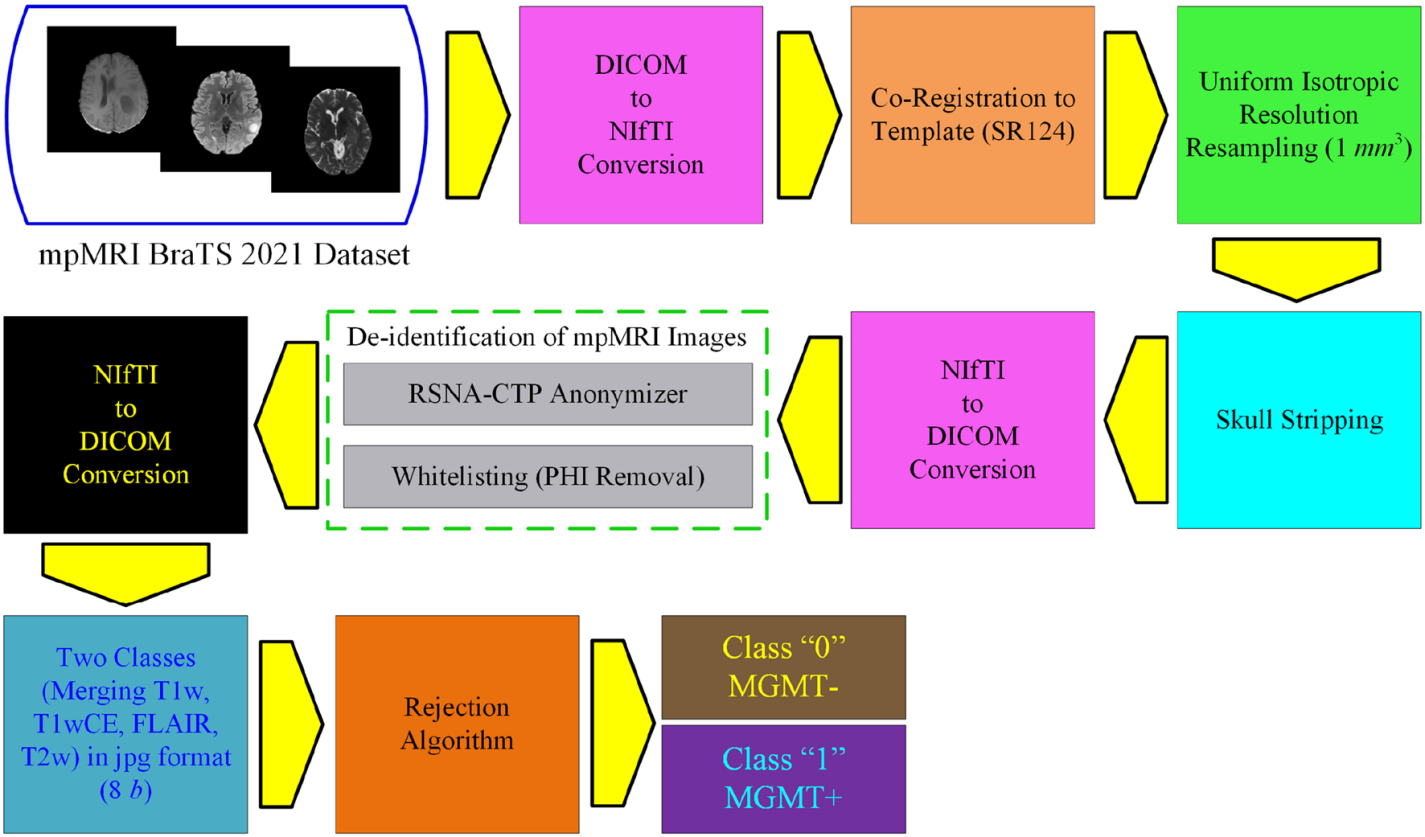
Efficient preprocessing steps for BraTS-2021 for mpMRI scans to MGMT methylation prediction stage.

Standardized preprocessing procedures have been adopted for all the mpMRI scans included in the BraTS-2021 dataset. The pre-processing included NIfTI file format conversion^58^, co-registration to the template of normal adult human brain anatomy for an MRI-based atlas SRI24^59^, resampling to 1*mm*^3^ isotropic resolution, followed by skull stripping, isolating cortex and cerebellum from the skull and nonbrain area, as illustrated in Fig. 3. All the preprocessing phases are handled using publicly available support such as Cancer Imaging Phenomics Toolkit (CaPTk) including Federated Tumor Segmentation (FeTS) tool^18,60–62^.

The preprocessing channel commenced with the conversion of the DICOM file format to NIfTI files^18,58^. The files for radiogenomic classification, one of the two tasks of the BraTS-2021 dataset, are not co-registered while for segmentation, the second task, the dataset undergoes registration to a standard template provided as NIfTI files. The NIfTI format removes the associated metadata including all the Protected Health Information (PHI) from the DICOM files. In addition, since the skull-stripping lessens the extent of facial reconstruction, which may be used for face recognition of the patient subsequently, it is based on a representation learning-based methodology that describes the brain shape prior and is independent of the MRI input^63–65^.

For radiogenomic classification, the entire mpMRI images are preprocessed as illustrated in Fig. 3, to generate the skull-stripped volumes, and finally converted from NIfTI to DICOM format consisting of skull-stripped images. Finally, a two-step process for data deidentification, comprising of RSNA CTP (Clinical Trials Processor) anonymizer^18^, and whitelisting of these outcome DICOM files, is carried out. This removes all unnecessary tags, ensuring the removal of all PHI entries, from the DICOM headers.

For radiogenomic classification, the entire images are segregated into two classes, namely “0” and “1” based on the presence of MGMT promotor methylation status, with T1W, T1WCE, FLAIR, and T2W combined in either of the classes depending on the annotation allocated by the expert radiologists and pathologists. These images are further converted from DICOM (12 bits) to jpg (8 bits) format and resized from 512 × 512 to 64 × 64 to speed up the training process, which is unavoidable for an enormous dataset, and takes care of memory issues encountered when using an average-price GPU based portable system.

### Pseudocode for rejection algorithm

**Figure 4:**
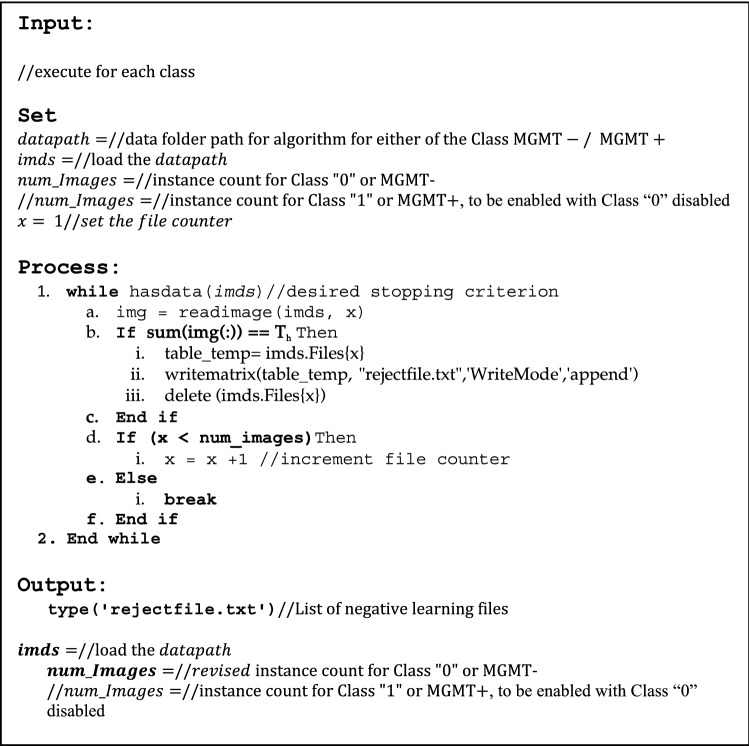
Pseudocode for the file rejection algorithm (RA).

The rejection algorithm, illustrated in Fig. 4, has been run for each class. The sole objective is to remove the instances that do not add discrimination to the radiogenomic classification process. The “datapath” of either of the binary classes is followed by exclusive loading of the entire image set for either of the classes, MGMT− (Class “0”) or MGMT+ (Class “1”). The number of images is fetched to act as the stopping criterion for the algorithm. The rejection algorithm checks the redundant/irrelevant images and removes them one by one from the specific class by using the threshold of the sum of pixel values (T_h_). After thorough investigation, T_h_ has been empirically found equal to zero for the optimum results. Once all the images have gone through RA, the output consists of the details for each of the rejected files, with the revised datapath loading producing the improved class details. The difference in the initial instance count of a class should match the rejected files count plus the final class size (after RA). The RA resulted in 26.75% and 27.51% reduction in the number of instances for classes “0” and “1” respectively.

### Deep learning-based latent shape features

**Figure 5:**
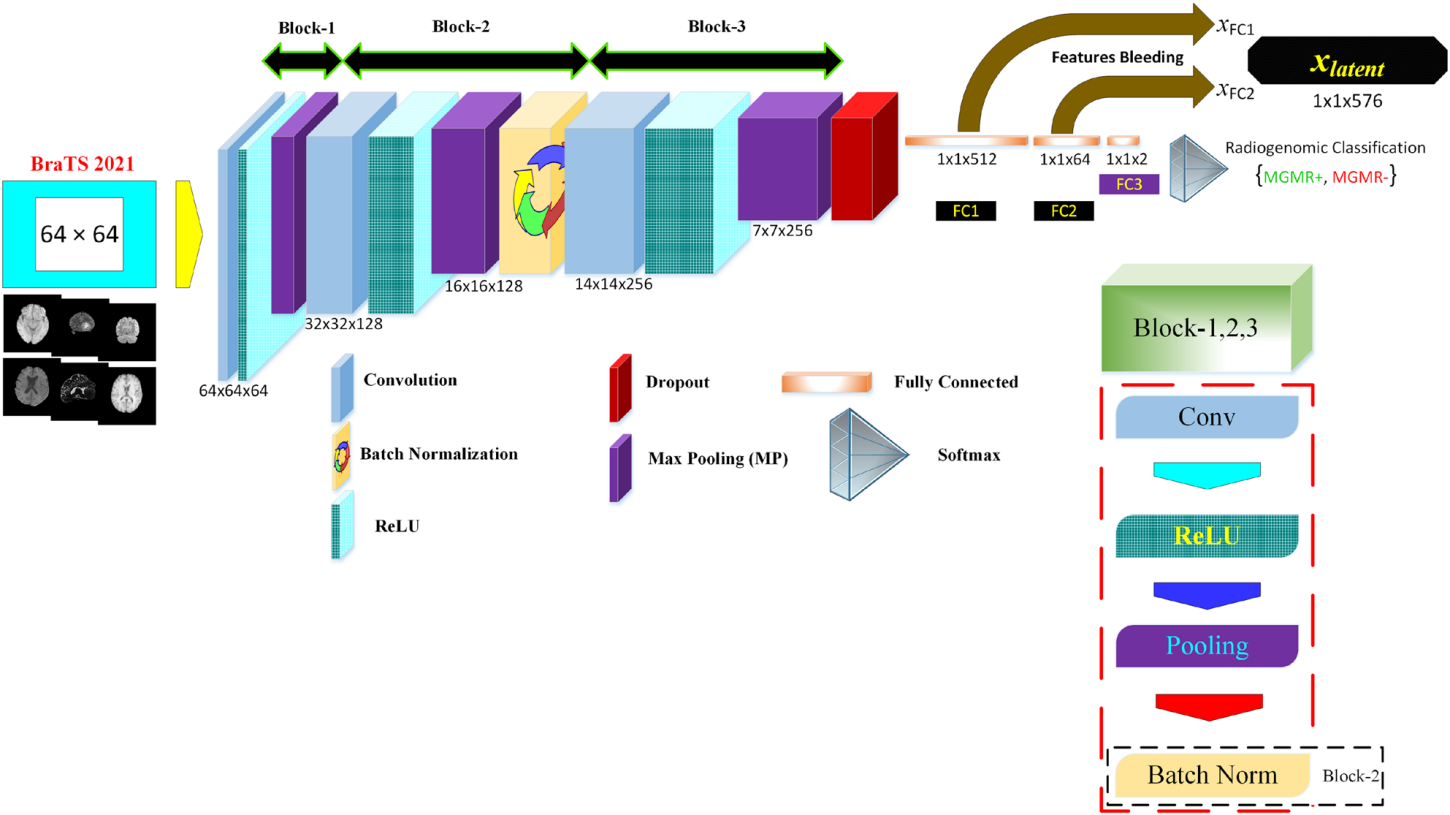
Latent feature extraction ${x}_{latent}$ using DLRFE-module via feature-bleeding through fully connected layers.

We propose a DLRFE module for radiomic characteristics using deep learning by feature bleeding through the fully connected layers. The parametric tuning of the module is carried out for the least memory requirements and computational overhead culminating in maximum efficiency using average-price GPU-based computer systems. It is composed of 3 convolution blocks as illustrated in Fig. 5. The weights and biases have been maintained at smaller values using L2-regularization and a dropout layer after the third block paving an improved generalization policy.

The DLRFE module is constituted of two networks; an encoder, to infer latent variables given input image, and a fully connected network to infer the prediction of the deep learning architecture. The input to the encoder network is an image sized: (64 × 64), and the output is a classification prediction. The encoder network consists of 3 convolutional network blocks (Block-1, Block-2, and Block-3) followed by three fully connected layers. Each block consists of convolutional, ReLU, and max-pooling layers with batch normalization after Block-2, and a dropout layer after Block-3.

The fully connected layer, after converting feature maps to a 1D vector, connects neurons to other layers, causing dynamic extraction of latent features. Three fully-connected layers in the DLRFE module are FC1, FC2, and FC3 with 512, 64, and 2 neurons respectively. The activation function is carried out by the SoftMax layer for the non-normalized output of FC3 using two neurons with a probability distribution in the range [0, 1] to a binary basis. The *k*th class probability as the cross-entropy loss (CEL) is determined using a normalized exponential function: ${P}_{k}=\frac{{e}^{{O}_{k}}}{\sum_{m=1}^{{\varvec{M}}}{e}^{{O}_{m}}}$ where *O*_k_ is the activation of the* k*th class. The CEL has two label sets: the target and expected labels are represented as *t*(*x*) and *p*(*x*) respectively. The loss is defined as: $L\left(t,p\right)=-\sum_{\forall x}t\left(x\right)\mathrm{log}\left(p\left(x\right)\right)$. The updating of weights during backpropagation is carried out by minimization of the cost function given by: $-\frac{1}{\mathrm{J}}\sum_{j=1}^{\mathrm{J}}ln\left(p\left({t}^{j}|{x}^{j}\right)\right)$, where J is the size of the training set, *x*^j^ represents the training sample with the target label *t*^j^ and *p*(*t*^j^|*x*^j^) is the classification probability. The stochastic gradient descent approach is used epoch-wise, with each epoch divided in the form of mini-batches, for the minimization of the cost function. The updated weight for the *i* + 1th iteration in layer *l*,${W}_{l}^{i+1}$ is given by: ${W}_{l}^{i+1}={W}_{l}^{i}+\Delta {W}_{l}^{i+1}$, where $\Delta {W}_{l}^{i+1}$ is the corresponding weight update. The network training takes place in a circular way, feed-forward, and feed-backward iteratively.

### Radiomic and radiogenomic features

Radiogenomics is based on quantitative data collected from medical images having individual genomic phenotypes, and the notion is to design a prediction framework to categorize patients for clinical outcomes. The genomic fractional variation in the tumor DNA can be obtained by multiple radiomic features during radiogenomic analysis using mpMRI scans^18,66,67^. The BraTS-2021 dataset for radiogenomic classification task is based on mpMRI scans and MGMT promoter methylation status. Many cohorts are employing this dataset leading to machine learning-based solutions for the prediction of MGMT promoter methylation status using radiomic features based on gray leveled imaging. Numerous radiomic feature extraction methods have been introduced and exploited depending on the nature of the problem structure and the corresponding solution domain. We have selected second-order statistics and textural features to build HFS in addition to the latent shape features ("Deep Learning-based Latent Shape Features"). We have used the three most effective feature extraction modules (FEMs) for radiogenomic classification tasks corresponding to individual feature extraction strategies, namely GLCM, HOG, and LBP. Each of the modules extracts diverse features independently from each of the mpMRI scans.

#### GLCM features

Many researchers have employed textural features successfully and solved classification-linked problems^68,69^, including the categorization of brain tumors^40,70^. We used GLCM-based FEM which is based on the spatial relationship of pixels in an image. These highly discriminative features describe the texture based on the repeatability of pixel groups with specific values that exist in a two-dimensional relationship in an image^71,72^. The GLCM is a square matrix (size: N × N), where N represents a different number of gray levels in an image. If the matrix is denoted by M(*i*, *j*) where each element (*i*, *j*) of GLCM represents the frequency of occurrence of a particular relationship between two intensities of pixels in the input image, where “*i*” represents the gray level of the pixel at location (*x*, *y*), and “*j*” represents the gray level of a contiguous pixel positioned at a relative distance “*d*” from the pixel at an orientation “*θ*”’. The relationship between the *i*th and *j*th intensities is based on these two parameters, *d* and *θ,* in four directions (0°, 45°, 90°, 135°) with an increment of 45° without symmetry repetition. We selected 13 Haralick features in our work as illustrated in Table 3 showing mathematical formulae for their calculation and comprehensive definitions^73^. These features are computed to form M, whereas ${p}_{ij}$ is (*i*, *j*)th entity of M divided by its size, the expressions m_i_ and m_j_, and σ_i_ and σ_j_ represent the mean and standard deviations of *i*th row and *j*th column of M. The directions averaged features are values are fused to form the GLCM-based feature vector ${x}_{GLCM}={[{F}_{1} {F}_{2} {F}_{3}\dots {F}_{13}]}^{t}$.

**Table 3:** Textural features used in the formation of HFS.

Features	Equation	Definition
Angular second moment	${F}_{1}=\sum_{i}\sum_{j}{ \left\{p(i,j)\right\}}^{2}$	A measure of uniformity of distribution of grey levels in the image
Contrast	$$F_{2} = \sum\limits_{{n = 0}}^{{N_{{g - 1}} }} {n^{2} } \left\{ {\sum\limits_{{\begin{array}{*{20}c} {i = 1} \\ {\left| {i - j} \right| = 1} \\ \end{array} }}^{{N_{g} }} {} \sum\limits_{{j = 1}}^{{N_{g} }} p (i,j)} \right\}$$	A measure of the local variations present in an image
Correlation	${F}_{3}= \frac{{\sum }_{i}\sum_{j}\left(ij\right)p\left(i,j\right)- {\mu }_{x}{\mu }_{y}}{{\sigma }_{x}{\sigma }_{y}}$	A measure of image linearity. It will be high if an image contains a considerable amount of linear structure
Variance	${F}_{4}=\sum_{i}\sum_{j}(i-{\mu }^{2})p(i,j)$	The variance is a measure of the dispersion of the gray-level differences at a certain distance
Inverse difference moment	${F}_{5}=\sum_{i}\sum_{j}\frac{1}{1+{\left(i-j\right)}^{2}}p(i,j)$	The measure of closeness of the distribution of GLCM elements to the GLCM diagonal
Sum Average	${F}_{6}=\sum_{i=2}^{{2N}_{g}}{ip}_{x+y}(i)$	The average sum of gray levels
Sum Variance	${F}_{7}= \sum_{i=2}^{{2N}_{g}}{\left(i-{F}_{8}\right)}^{2}{p}_{x+y}(i)$	The variance of a sum of gray levels
Sum entropy	${F}_{8}= -\sum_{i-2}^{{2N}_{g}}{p}_{x+y}\left(i\right)\mathrm{log}\left\{{p}_{x+y}(i)\right\}$	The uniform distribution of a sum of gray levels has maximum entropy
Entropy	${F}_{9}=-\sum_{i}\sum_{j}p(i,j)\mathrm{log}(p(i,j))$	A measure of information content
Difference variance	${F}_{10} = {\text{variance}} \,\text{of}\, {p}_{x-y}$	The variance of a difference in gray levels
Difference entropy	${F}_{11}= -\sum_{i=0}^{{N}_{g-1}}{p}_{x-y}(i)\mathrm{log}\left\{{p}_{x-y}(i)\right\}$	The uniform distribution of a difference in gray levels has maximum entropy
Information measures of correlation 1	${F}_{12}=\frac{HXY-HXY1}{max\left\{HX,HY\right\}}$	Normalized mutual information
Information measures of correlation 2	${F}_{13}={(1-exp\left[-2.0(HXY2-HXY)\right])}^{1/2}$ $HXY= -\sum_{i}\sum_{j}p(i,j)\mathrm{log}(p(i,j))$ $HXY1= -\sum_{i}\sum_{j}p(i,j)\mathrm{log}\left\{{p}_{x}(i){p}_{y}(j)\right\}$ $HXY2= -\sum_{i}\sum_{j}{p}_{x}(i){p}_{y}(j)\mathrm{log}\left\{{p}_{x}(i){p}_{y}(j)\right\}$	The difference between joint entropy and joint entropy assuming independence

**Figure 6:**
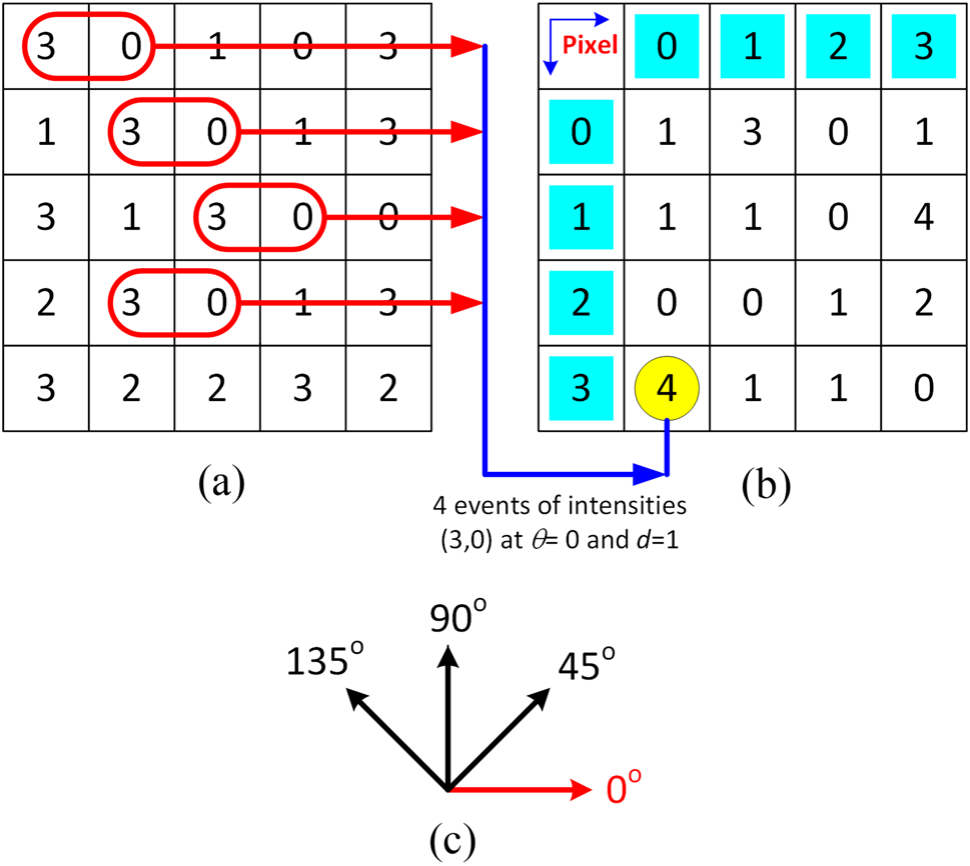
A simple diagram for GLCM computation; (**a**) input matrix, (**b**) GLCM computed from (**a**) using *d* = 1 at 0° orientation, and (**c**) set of orientation, *θ*.

A schematic diagram for GLCM calculation has been shown in Fig. 6. In the GLCM illustration, Fig. 6b, the top row and left column, cyan cells, are pixels presented in the input matrix. The GLCM, Fig. 6b, calculation using the input matrix, Fig. 6a, illustrates the calculation of the matrix at 0° and $d=1$ for neighboring intensity pairs (3,0). In Fig. 6a, a pixel with intensity ‘3’ is present with pixel ‘0’ in a pair (3,0) four times as shown with a yellow circle in Fig. 6b. Similarly, the entire GLCM is calculated for single orientation and adjacent pixel intensities pairs.

#### HOG features

**Figure 7:**
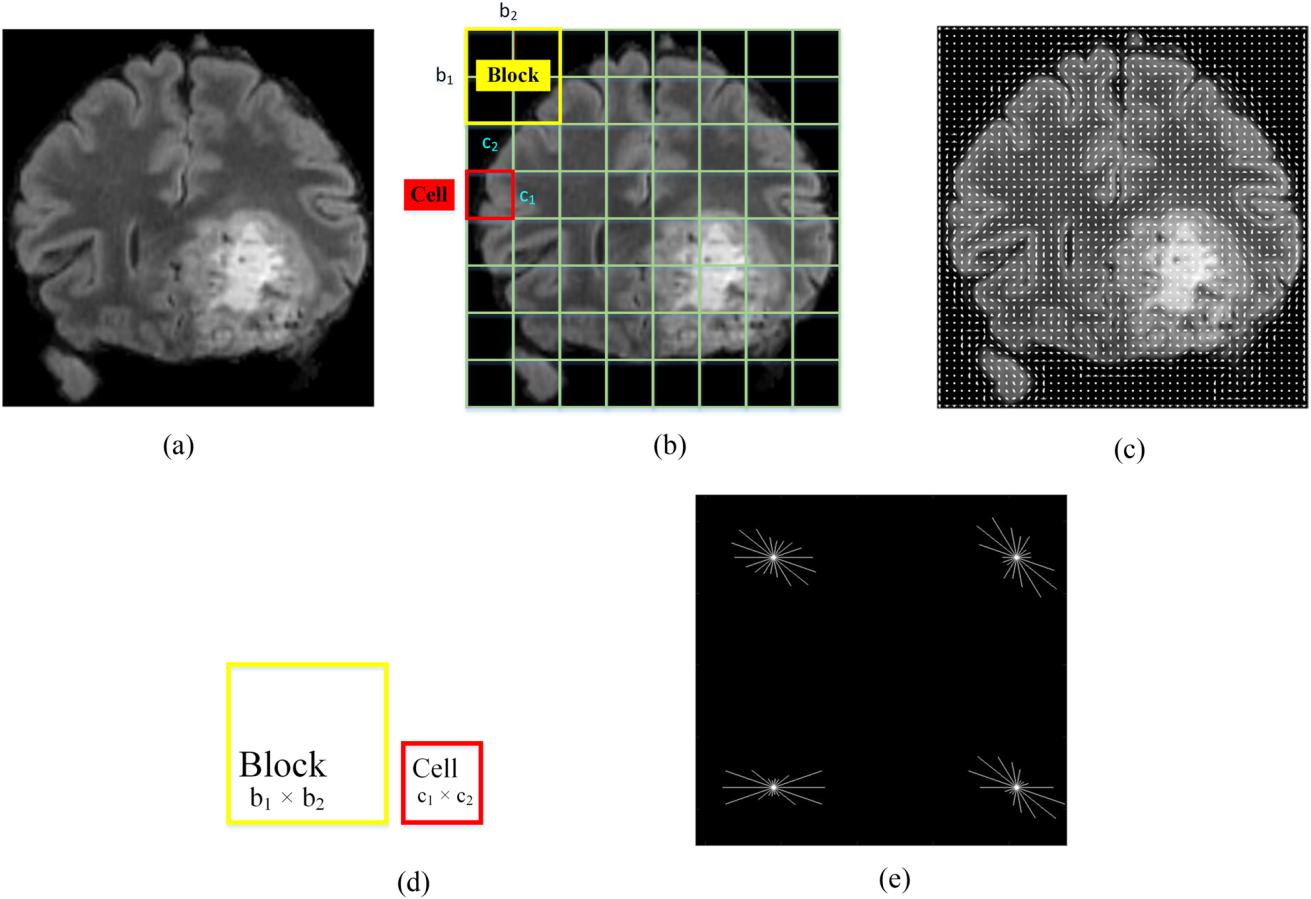
Schematic diagram of FEM for HOG features; (**a**) a rescaled mpMRI scan for radiogenomic dataset, (**b**) schematic HOG cells and blocks superimposed on (**a**), (**c**) HOG descriptors, (**d**) cell and block representation, and (**e**) the magnified view of a single cell.

HOG features efficiently perform classification tasks due to highly discriminative characteristics associated with their extraction procedure^74,75^. The HOG module extracts the direction and gradient-based information from the input image that helps in describing the structure of the problem. The HOG feature extraction process is illustrated in Fig. 7a–e. Figure 7a shows an original mpMRI scan (BraTS-2021 dataset), while Fig. 7b represents the schematic of cells and a block superimposed on the original input image. Figure 7c shows the HOG descriptors with a schematic of a block and a cell, both depicted separately in Fig. 7d, while the magnified view of a single cell is shown in Fig. 7e. The feature extraction strategy consists of three activities:

i. The computation of gradients by calculating the direction or magnitude of each pixel. The Sobel kernel function is used to obtain gradient in ${E}_{x}$ and ${E}_{y}$ directions to calculate gradient and angle at every pixel using the mathematical formulation: ${M}_{HOG\left|g(i,j)\right|}= \sqrt{{E}_{x}({i,j)}^{2}+{E}_{y}({i,j)}^{2}}$, and ${M}_{{HOG}_{\theta g(i,j)}}={\mathrm{tan}}^{-1}\left(\frac{{E}_{y}(i,j)}{{E}_{x}(i,j)}\right)$, where, $\left|g(.)\right|$ denotes magnitude, ${\theta }_{g }(.)$ denotes the direction of the gradient, *i* and *j* denote rows and columns respectively.
ii. The small cells of size (r × s) are derived from the input image as shown schematically in Fig. 7b,d.
iii. Finally, the cells are combined into overlapping blocks, each of size (p × q), and in each block, a histogram of oriented gradients falling into each bin is computed which is further subjected to the normalization process (L_2_-norm) to overcome illumination variation. The normalized vector for each block of the histogram is given by: ${M}_{HOG{T}_{i}^{N}}=\frac{{T}_{i}}{\sqrt{{{T}_{2}}^{2}+{\in }^{2}}}$, where $\in$ is any constant that can’t be divided by zero, and $T$ indicates the non-normalized vector.

The features so collected from all the normalized blocks are fused to form a feature descriptor, *x*_HOG_, for the entire image.

#### LBP features

**Figure 8:**
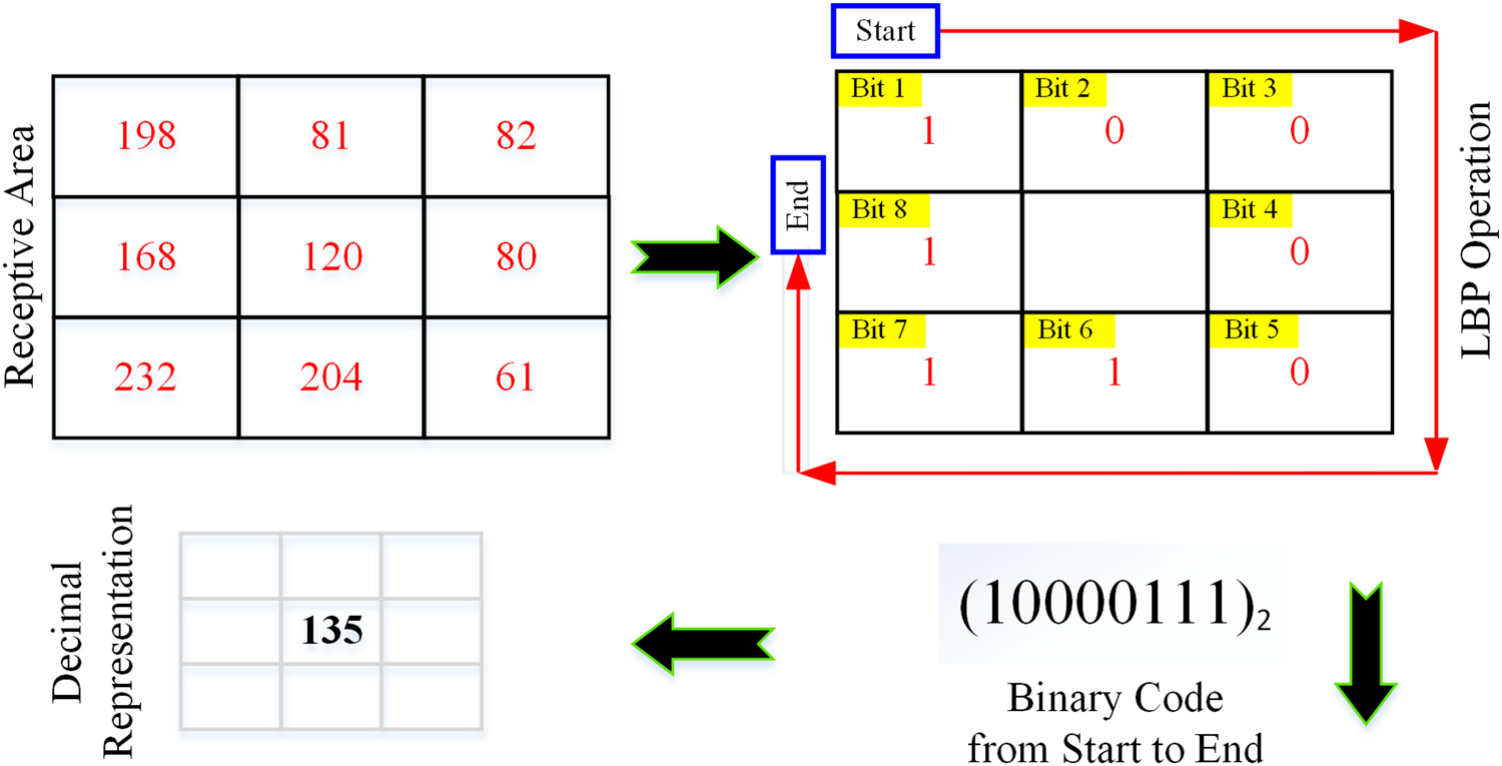
Schematic diagram of LBP operation to find the discriminative features.

Local binary patterns (LBP) descriptor is another efficient feature extraction operator generating high-level characteristics that are used to assign a label to each pixel of an image by thresholding its neighborhood and translating the result as a binary number^76^. LBP has the advantages of rotation invariance along with gray level invariance^77^. This feature module encodes the relationship between the pixel and its neighbors in a circular manner by describing the local spatial structure of an image. The binary output using the LBP operator is obtained using the difference (G_p_- G_c_ ) on a per-pixel basis and checking it through the central pixel (G_c_) along with the surrounding pixels G_p_, where *p* is limited in the range^1,8^ around 3 × 3 receptive areas^30^. The working principle of the LBP operator on a pixel is illustrated in Fig. 8. The LBP values are computed by binarization based on the difference between the contiguous pixels, where two groups are formed and each element is assigned to either of the group, with the help of a step function. The central pixel value ${LBP}_{p,r}({G}_{c})$ is given by:

1 $${LBP}_{p,r}({G}_{c})= \sum_{p=0}^{p-1}H\left({G}_{p}-{G}_{c}\right){2}^{p} , H\left(x\right)=\left\{\begin{array}{l}1\quad \, x\ge 0\\ 0\quad \,otherwise\end{array}\right.$$

The threshold function, H(x), treats values that are greater than zero or equal to zero. In the above equation, *r* represents the central pixel distance from the neighboring pixels (radius), and *p* represents the total number of pixels minus the central pixel included in the process^78,79^. In Fig. 8, *r* = 1 and *p* = ^1,8^, have been employed with a receptive area of 3 × 3 sized mini-image. The last stage is carried out by converting the binary codes of zeros and ones to decimal numbers to form an LBP image^80^.

### Jackknife cross-validation

The Jackknife cross-validation with tenfold engaged in this work for parametric optimization including the training and test data formulation. It is a commonly used approach for the verification of robustness and confidence of the system performance (Fig. 1) for model selection on potential algorithms^81^. In this technique, the data is divided into 10 folds (${F}_{i};i=\mathrm{1,2},\dots 10)$ or partitions. The test portion, based on a single fold, is crossed and the training portion consisting of (K-1) folds is partitioned into training and validation sets^82^. The diagonal folds, shown as crossed rectangles (${F}_{i}{A}_{i};i=\mathrm{1,2}, \dots ,10$), represent the test partitions and the off-diagonal folds, non-cross folds, represent training partitions, shown as plain rectangles (${F}_{i}{A}_{j};i=\mathrm{1,2}, \dots ,10; j=\mathrm{1,2}, \dots ,10 \, AND\, i\ne j$), which are divided into training and validation sets. The best parameters-based model is used with the test data to evaluate the performance. We have used the stratified cross-validation scheme due to the imbalanced class distribution in the dataset to give a close approximation of the generalization accuracy (${A}_{i};i=\mathrm{1,2},\dots ,10$). This ensures an equal number of instances of each class distributed across the training and test partitions^83^. The performance (***A***_***c***_) estimated by cross-validation using each test fold accuracy A_*i*_  ∈  $\Re$ *i* = 1, 2, 3,…, K, folds is given by:

2 $${{\varvec{A}}}_{{\varvec{c}}}=\overline{A }\pm \sqrt{\frac{\sum_{i=1}^{K}{\left({ A}_{i}-\overline{A } \right)}^{2}}{K-1}}$$

The average performance $\overline{A }$ is given by:

3 $$\overline{A }=\frac{1}{K}\sum_{i=1}^{K}{A}_{i}$$

### Classification model

The individual features including their combinatorial hybrids and HFS have been employed in the classification system using SVM and *k*-NN algorithms as the potential ML tools to find a reliable classification solution. The SVM was optimized using linear, RBF, and polynomial kernels. A brief overview of the classifiers being selected for this article is given by:

#### *k*-Nearest neighbors (*k*-NN) classification

The *k*-NN, a proximity-based classifier, is based on the notion that the test instance *q* would have the maximum number of nearest neighbors for the prospective class. In this classification technique, distance *d* is measured from query *q* to k-nearest neighbors, *x*^t^ that lie in class *y*^t^. The q is assigned the class *y*^u^ having the maximum number of neighbors among the k-nearest neighbors^84,85^. In the case of $k=1$, the test instance is simply assigned the class of the single nearest neighbor. The formula for Minkowski distance ${d}_{mk}$, a generalized distance metric is given by:

4 $${d}_{mk}\left(q, {x}^{t}\right)={\left(\sum_{t=1}^{k}{\left|q-{x}^{t}\right|}^{r}\right)}^\frac{1}{r},$$

where the larger value of *r* gives more influence to the distance on which *q* differs the most. Some of the distance metrics are Euclidean (L_1_-norm), Mahalanobis (L_2_-norm), and Chi-square distances as given in Table 4. Another variant mostly used is distance weighted-voting where the *q* receives vote *V* from each of the nearest neighbors weighted inversely to their distance from *q*^85^:

5 $$V\left({y}^{u}\right)=\sum_{t=1}^{k}\frac{1}{d{\left(q,{x}^{t}\right)}^{z}} 1\left({y}^{u},{y}^{t}\right),$$

where $1\left({y}^{u},{y}^{t}\right)$ is unity when both labels match, zero otherwise, and *z* determines the type of distance measure being adopted during classification.

**Table 4:** Distance functions for *k*-NN classifier.

Distance	Function
Euclidean	${d}_{e}=\sqrt{\left(\frac{{\sum }_{t=1}^{k}{\left(q-{x}^{t}\right)}^{2})}{k}\right)}$
Chi-square	${d}_{c-s}={\underset{ }{\mathit{max}}}_{j}(\left|q-{x}^{t}\right|)$
Mahalanobis	${d}_{m}={\left[{\left(q-{x}^{t}\right)}^{T}{\Sigma }^{-1}\left(q-{x}^{t}\right)\right]}^{1/2}$

#### Support vector machine classification

The SVM maps the supervised-learning *t*th instance pair (*x*^*t*^*, y*^*t*^), *x*^*t*^ being the sample with label *y*^*t*^, from a data set *X* in the sample space *S* separated by a hyperplane with a maximized margin on either of its sides^86^. The class label-based least-confident points near the hyperplane are the support vectors. The better generalization of the model depends on maintaining the margin as much as possible. The outlier (noise) does not influence the decision boundaries significantly as it would simply ignore its effect in the training phase^87^. On the other hand, the SoftMax layer in CNN will be influenced by such a point due to its probability-based working principle. In other words, the SVM is favored as a strong classifier with a reduced error rate. The hyperplane function, defined for the linearly-separable classes, is given for the *i*th class by:

6 $${g}_{i}\left(\underline{x}\right)={\mathcalligra{w}}_{i}^{T}\underline{x}+b {\mathcalligra{w}}\in S, b\in {\mathbb{R}},$$

where $\underline{x}$ is the input training vector and ${\mathcalligra{w}}_{i}^{T}$ is weight vector that is orthogonal to the hyperplane for ${i}$th class, and $b$ is the bias of the decision plane. The distance on both sides of the hyperplane defines a margin that is maximized when the weight vector $\Vert {\mathcalligra{w}}\Vert$ is minimum. To find the optimal separating hyperplane, SVM aims to maximize the margin as given by:

7 $$\begin{aligned}&\mathrm{min}\frac{1}{2}{\Vert {\mathcalligra{w}}\Vert }^{2},\nonumber\\&{\text{subject to:}}\nonumber\\ &{\mathcalligra{w}}{x}^{t}+b\left\{\begin{array}{l}\ge 1\quad \, for \, {y}^{t}=1,\\ otherwise\quad \, {y}^{t}= -1,\end{array}\right. \quad \forall t {x}^{t}\in {\mathbb{R}}^{m},{y}^{t}\in \left\{+1,-1\right\}.\end{aligned}$$

### Performance measures

The evaluation of the model is established on performance measures like confusion matrix, accuracy, sensitivity, specificity, precision, negative predictive value, receiver operator characteristic curve curves, F_1_-score, and Matthews correlation coefficient. “*TP*” is the number of images detected with a specific promoter methylation status and actually, they are of the same status. “*FN*”, also known as *Type II error*, is the number of images detected not found with a specific promoter methylation status but truly they are having that specific status and “*FP*”, also known as *Type I error*, is the number of images detected with a specific promoter methylation status but they are not truly having that status. “*TN*” is the number of images that neither are having a specific promoter methylation status nor are labeled as having a specific status by the classifier.

**Accuracy**, *A*, defines the effectiveness of a classification model as a quantitative measure. It can be calculated by using the following relationship: $A=\left(TP+TN\right)/\left(TP+FP+FN+TN\right) \times 100$.

**Sensitivity**, S_n_, defines the usefulness of a model to know instances of MGMT+ class. It can be computed by: ${S}_{n}=TP/\left(TP+FN\right)$. This measure of performance is also known as Recall (R_c_) or true positive rate (TPR).

**Specificity**, S_p_, defines the usefulness of a classifier to know instances of MGMT− class. It can be computed by: ${S}_{p}=TN/\left(TN+FP\right)$. The specificity is also known as true negative rate (TNR).

**Precision**, P_r_, defines the proportion of all cases testing positives that truly belong to MGMT+ class. It can be calculated by: ${P}_{r}=TP/\left(TP+FP\right)$. The precision is also known as positive predictive value (PPV).

**Negative predictive value**, NPV, defines the proportion of all cases testing negatives that truly belong to MGMT− class. It can be calculated by: $NPV=TN/\left(TN+FN\right)$.

***F***_***1***_**-score**, measured in the range [0, 1], is the harmonic mean of P_r_ and R_c_, and it can be mathematically expressed as: ${F}_{1}-score=\left(2\times {P}_{r}\times {R}_{c}\right)/\left({P}_{r}+{R}_{c}\right)$. This measure is significant in case the class imbalance is present in the dataset.

**Mathews correlation coefficient** (MCC) is considered important as a performance measure in the case of binary classification problems. It varies in the range [− 1, + 1], where the value + 1 indicates the right predictive decision in agreement with the label, − 1 indicates the misclassified prediction, and 0 indicates the random prediction with 50% accuracy. Mathematically, MCC can be calculated by: $MCC=\left(TP\times TN-FP\times FN\right)/\sqrt{\left(TP+FN\right)\left(TN+FN\right)\left(TP+FP\right)\left(TN+FP\right)}$.

**Receiver operating characteristic** (ROC) curve defines the classifier’s overall performance over the entire operating range depicting the classification efficiency. TPR represents S_n_, whereas FPR is defined as the count of MGMT− events expected as MGMT+ events divided by the total sum of MGMT− events^88^. The mathematical formulation of FPR is given by: $FPR=FP/\left(TN+FP\right)$.

### Ethical approval

All the procedures were performed in accordance with the relevant guidelines and regulations.

## Results and discussion

We analyzed the proposed framework to categorize mpMRI scans into either MGMT+ or MGMT− instances. After preprocessing, GB images are used for latent and radiomic features extraction, feature fusion is investigated and HFS so formed is forwarded to a strong classification algorithm. All of the experiments have been conducted using open source libraries, and the standard programming tools used to tune the proposed system using Dell G7 Laptop (Intel® Core™ i7 8th Generation CPU), 32 GB RAM, and 6 GB GPU (NVIDIA GTX-1060 and 1280 CUDA cores).

### Experimental setup

The setup was initiated by selecting appropriate parametric values to extract the latent and radiomic features. The experimentation has been carried out by employing the tenfold Jack-knife cross-validation on the BraTS-2021 dataset. The optimal heuristics selection has been discussed in "Selection of optimal parameters". The contrast normalization was carried out in the range [0–1] before the classification stage. The classification results ("Dataset") have been generated on unseen test samples, with hidden labels used only for performance measurement, of the test folds. Sections "Performance analysis of DL-based latent feature extraction", "Analysis of parameters for Deep learning and radiomic feature methods", and "Performance analysis of individual FEMs" analyze the classification performance of the latent-, individual- and hybrid-features respectively. Section "Performance analysis of HFS" illustrates the time involved in feature extraction (two stages), and finally classification during the training and testing phases. Section "Performance comparison" describes a comprehensive comparison of the proposed technique with existing schemes in terms of classification performance followed by the shortcomings and the future recommendations for cohorts working in the areas of common interest.

### Selection of optimal parameters

The classification performance of our framework, the MGMT-PMP system, is dependent on the tuning of numerous parameters for classification using ML classifiers. Subsequently, the analysis of optimal values of the DLRFE module, latent feature extraction, radiomic feature modules, and the strong machine learning models have been presented in detail. Only *k*-NN and SVM classifiers have been used, and the parameterization has been achieved based on classification performance accuracy. We have used these optimal values in the forthcoming sections.

#### k-NN model

The proposed model has been experimented with four variants of the *k*-NN classifier ($k=\mathrm{1,3},\mathrm{5,7}$) to categorize HFS into MGMT+ and MGMT− classes. In our case, the results for the *k*-NN classifier have been found to outclass relatively at $k=1$ as illustrated in Table 5 for the BraTS-2021 dataset without RA. The performance of the *k*-NN model has been shown in Table 6 with RA.

**Table 5:** Proposed system results (tenfold-cross validated) with epochs variation depicting analysis-based performance using *k*-NN classifier with *k* = 1 for BraTS-2021 dataset (without RA).

Epoch	S_p_ (%)	R_c_ (or S_n_) (%)	P_r_ (or PPV) (%)	NPV (%)	AUC (ROC)	F_1_-score	MCC	A (%)
1	70.24 ± 0.01	96.57 ± 0.14	71.70 ± 0.03	96.33 ± 0.15	0.83 ± 0.00	0.82 ± 0.00	0.67 ± 0.00	81.78 ± 0.06
**5**	**70.48 ± 0.00**	**96.96 ± 0.00**	**71.94 ± 0.00**	**96.74 ± 0.00**	**0.84 ± 0.00**	**0.83 ± 0.00**	**0.68 ± 0.00**	**82.09 ± 0.00**
10	70.16 ± 0.00	96.50 ± 0.00	71.63 ± 0.00	96.25 ± 0.00	0.83 ± 0.00	0.82 ± 0.00	0.67 ± 0.00	81.71 ± 0.00
15	69.36 ± 0.15	95.41 ± 0.15	70.85 ± 0.13	95.09 ± 0.00	0.82 ± 0.00	0.81 ± 0.00	0.65 ± 0.00	80.78 ± 0.15
20	68.88 ± 0.65	94.81 ± 0.38	70.40 ± 0.35	94.44 ± 0.34	0.82 ± 0.00	0.81 ± 0.00	0.64 ± 0.00	80.25 ± 0.20
25	68.86 ± 0.26	94.67 ± 0.36	70.36 ± 0.10	94.31 ± 0.00	0.82 ± 0.00	0.81 ± 0.00	0.64 ± 0.00	80.18 ± 0.01
30	68.78 ± 0.32	94.24 ± 0.70	70.21 ± 0.06	93.86 ± 0.67	0.82 ± 0.00	0.80 ± 0.00	0.64 ± 0.00	79.94 ± 0.13
40	68.58 ± 0.27	93.89 ± 0.30	70.00 ± 0.12	93.50 ± 0.27	0.81 ± 0.00	0.80 ± 0.00	0.63 ± 0.00	79.68 ± 0.02
50	68.50 ± 0.05	93.98 ± 0.18	69.96 ± 0.00	93.58 ± 0.17	0.81 ± 0.00	0.80 ± 0.00	0.63 ± 0.00	79.67 ± 0.05

Significant values are given in bold.

**Table 6:** Proposed system results (tenfold-cross validated) with epochs variation depicting analysis based on performance using *k*-NN classifier with *k* = 1 for BraTS-2021 dataset (with RA).

Epoch	S_p_ (%)	R_c_ (or S_n_) (%)	P_r_ (or PPV) (%)	NPV (%)	AUC (ROC)	F_1_-score	MCC	A (%)
1	96.51 ± 0.16	95.26 ± 0.18	95.56 ± 0.20	96.29 ± 0.23	0.96 ± 0.00	0.95 ± 0.00	0.92 ± 0.00	95.96 ± 0.16
**5**	**97.08 ± 0.10**	**95.86 ± 0.14**	**96.29 ± 0.13**	**96.80 ± 0.13**	**0.96 ± 0.00**	**0.96 ± 0.00**	**0.93 ± 0.00**	**96.54 ± 0.10**
10	96.83 ± 0.00	95.02 ± 0.00	95.94 ± 0.00	96.10 ± 0.00	0.96 ± 0.00	0.95 ± 0.00	0.92 ± 0.00	96.03 ± 0.00
15	95.71 ± 0.31	93.94 ± 0.15	94.53 ± 0.37	95.17 ± 0.11	0.95 ± 0.00	0.94 ± 0.00	0.90 ± 0.00	94.93 ± 0.15
20	95.18 ± 0.01	93.12 ± 0.52	93.85 ± 0.04	94.48 ± 0.44	0.94 ± 0.00	0.93 ± 0.00	0.88 ± 0.00	94.27 ± 0.23
25	94.77 ± 0.22	92.98 ± 0.20	93.34 ± 0.26	94.48 ± 0.14	0.94 ± 0.00	0.93 ± 0.00	0.88 ± 0.00	93.89 ± 0.12
30	94.53 ± 0.15	92.97 ± 0.06	93.06 ± 0.17	94.46 ± 0.04	0.94 ± 0.00	0.93 ± 0.00	0.88 ± 0.00	93.84 ± 0.06
40	94.40 ± 0.49	92.24 ± 0.31	92.85 ± 0.55	93.91 ± 0.20	0.93 ± 0.00	0.93 ± 0.00	0.87 ± 0.00	93.45 ± 0.14
50	94.19 ± 0.53	92.49 ± 0.58	92.63 ± 0.58	94.08 ± 0.40	0.93 ± 0.00	0.93 ± 0.00	0.87 ± 0.00	93.44 ± 0.04

Significant values are given in bold.

**Figure 9:**
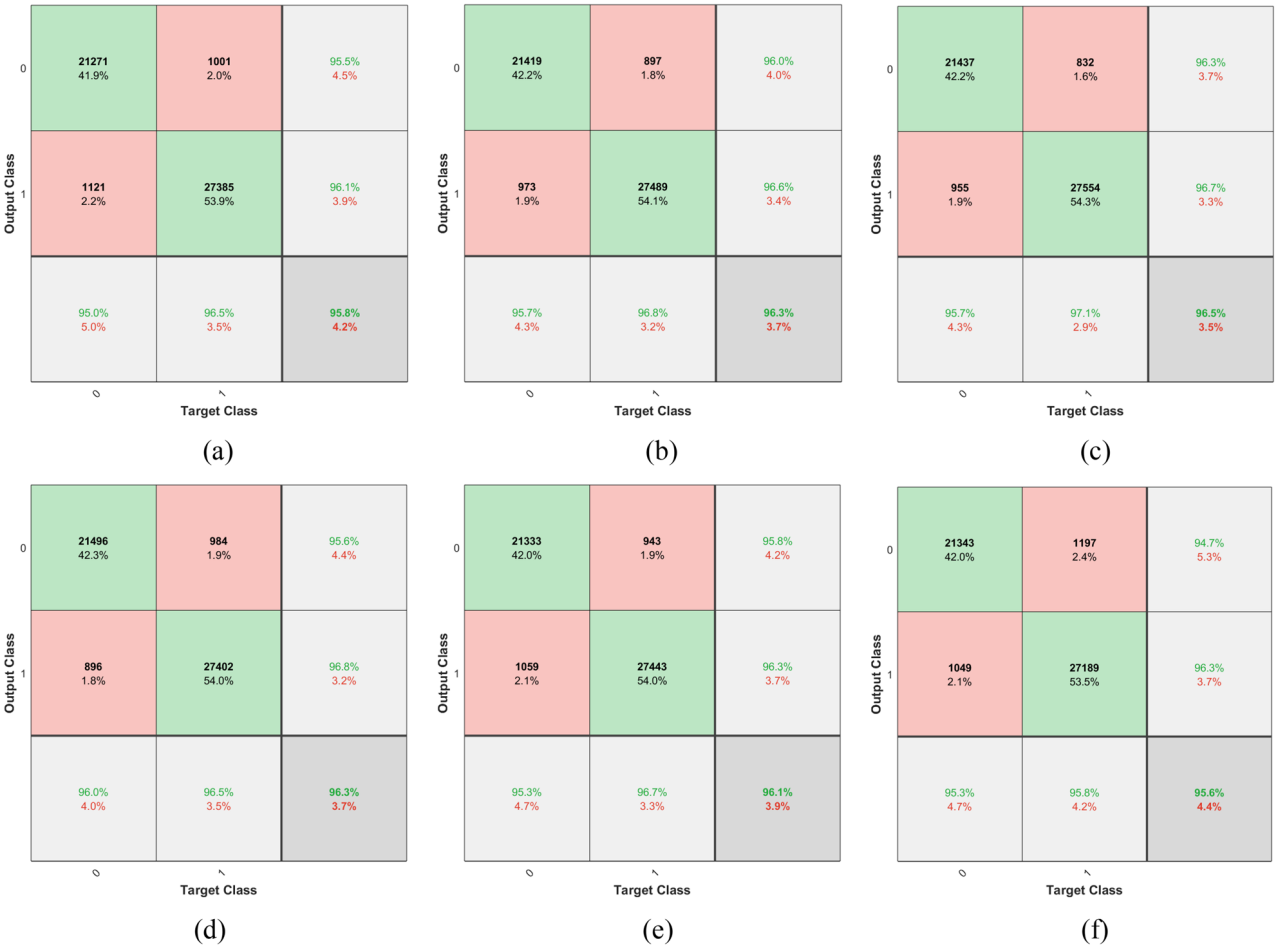
Confusion Matrices (based on the best of ten) for the *k*-NN classifier (*k* = 1) using proposed DLRFE module for backbone learning using BraTS-2021 dataset with RA to improve the discrimination of the features using epochs as: (**a**) 1, (**b**) 5, (**c**) 10, (**d**) 25, (**e**) 30, and (**f**) 50.

The confusion matrices for varying numbers of epochs have been illustrated in Fig. 9 for radiogenomic classification of the BraTS-2021 dataset with *k*-NN classifier with RA. The correlation between the features for the same class, resulted in the reduction of false events for test instances after the RA is applied to the original dataset. If we compare the classification results using Table 5, it is inferred that the application of RA resulted in improving the discriminative features (Fig. 6) thereby alleviating the characteristics that are based on similar or overlapping features. The confusion matrices in Fig. 6 show that excellent results have been found from higher to lower epoch counts as the deep learning module has been used alone for dynamic feature extraction, and the *k*-NN is engaged for training and test purpose using these features.

**Figure 10:**
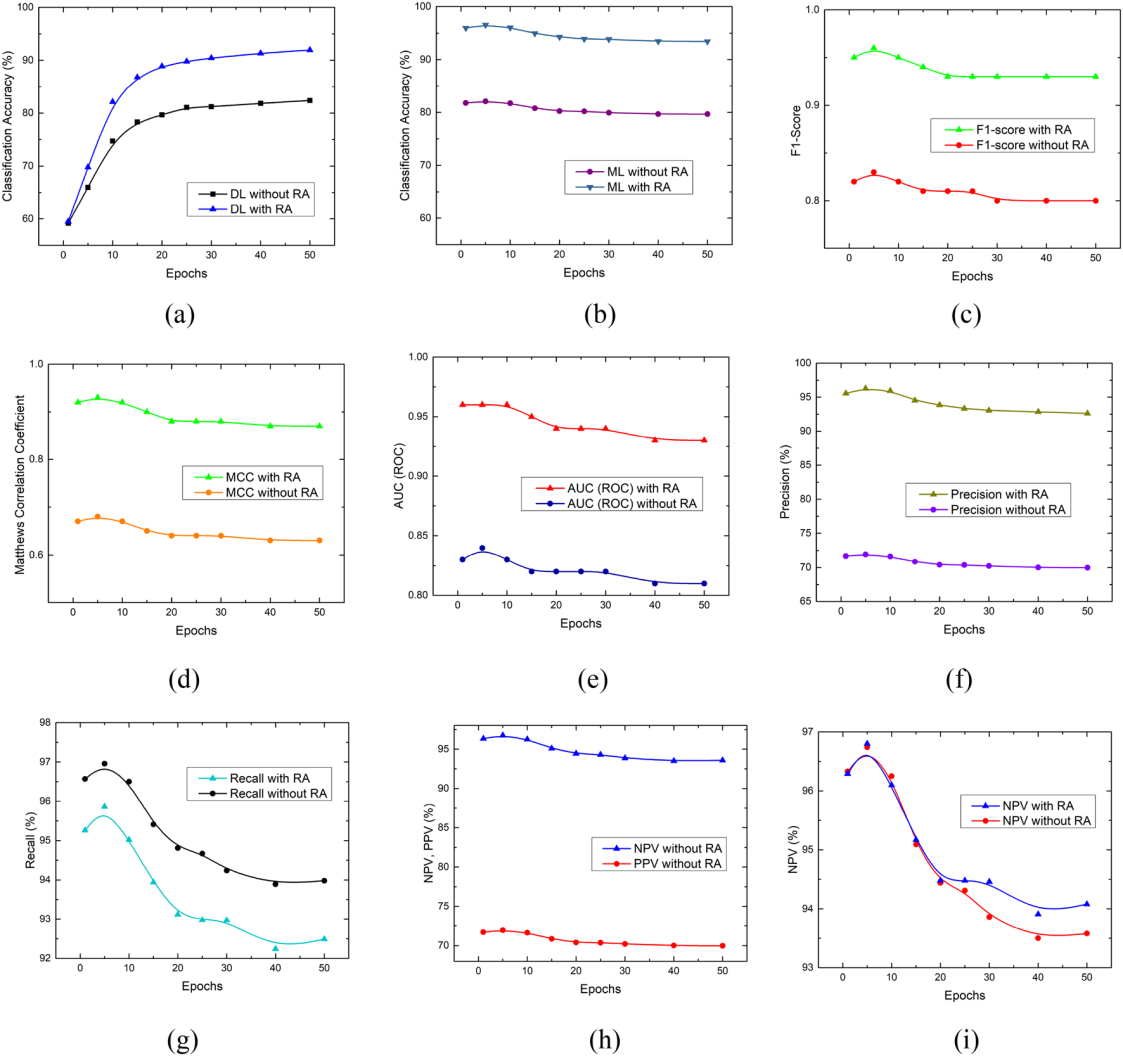
Performance analysis of RA for BraTS-2021 dataset based on: (**a**) accuracy variation with DL with softmax used as the classifier, (**b**) accuracy variation using *k*-NN model ML with *x*-axis showing the epochs used to retrieve the ${x}_{latent}$ (**c**) F_1_-score plot, (**d**) MCC plot, (**e**) AUC (ROC) variation with epochs, (**f**) precision variation with epochs, (**g**) recall variation with epochs, (**h**) PPV and NPV plots for varying epochs without RA, (**i**) NPV with and without RA plots.

The improvement in classification performance using RA has been found remarkable as shown in Fig. 10 which is a visual representation of quantitative analysis illustrated in Tables 5 and 6. Figure 10a explains the trends followed by a variation of training epochs employed by the DLRFE module with and without RA. Initially, the classification accuracy exhibits transitory behavior indicating a converging trend followed by a uniform behavior till the termination of 50 epochs. Figure 10b shows the ML-based classification accuracy using a *k*-NN model and latent features with and without RA. The plot represents epochs on the x-axis that have been used before the DL features were bled from the main architecture through the last fully connected layers ("Deep learning-based latent shape features"). It has been found that the classification accuracy is insensitive to the varying epochs so that the lesser epochs are sufficient to exploit the discriminative features derived for the proposed framework. F_1_-score measures reliable performance in case of imbalanced datasets, i.e. BraTS-2021. Figure 10c illustrates trends followed by the F_1_-score for original and RA-based datasets with the variation of training epochs for representation-learning-based discriminative features. It has been found that RA modified dataset resulted in outclass performance at its fifth epoch-based latent features. The low epoch features relate to the underfitting of the model due to high bias. These features resulted in good generalization during machine learning according to Occam's razor principle, stating the simplest solution is the best. The higher number of epochs corresponds to training even on noise distribution causing overfitting, and sacrificing generalization, so that the resulting model with high variance exhibits compromised performance on unknown instances. Figure 10d shows a similar trend for MCC indicating the performance improvement using RA for the original dataset. Figure 10e shows the AUC (ROC) plot showing the marked improvement with RA. Figure 10f follows the P_r_ trend indicating the performance rise due to RA. Figure 10g shows a similar trend of R_c_ with and without RA. A careful look at Table 5 indicates that the prediction discrimination threshold between positive and negative sides appears to increase S_n_, by moving towards the latter, reducing FNs as illustrated in Table 7 showing the cross-validated (tenfold) distribution of confusion matrix components for different epochs runs for latent features extraction. At the same time, S_p_ decreases due to increased FPs following the same logic. A similar trend is encountered between P_r_ and R_c_ as that of S_p_ and S_n_ respectively. There is a slight decrease in R_c_ with RA due to a slight increase in FN but at the same time has the effect of raising S_p_ from (70.48 ± 0.00)% to (97.08 ± 0.10)% using Tables 5 and 6 respectively at epochs = 5 with *k*-NN for *k* = 1. The overall effect of RA is a balanced increase in S_p_ and S_n_, i.e. (97.08 ± 0.10)% and (95.86 ± 0.14)% respectively as illustrated in Table 6 (*k*-NN with *k* = 1, epochs = 5). It can be inferred that high S_n_ (↓FNs) is accompanied by positive results and high S_p_ (↓FPs) leads to healthy subjects. Figure 10h shows the comparison of higher to lower NPV trends. This can be explained based on previous findings. Figure 10i plot has been based upon NPVs with and without RA. The latter has shifted the predictive threshold boundary to the negative side so that FNs are reduced. In the case of RA based NPV plot, the predictive threshold boundary between positive and negative subjects is balanced in such a way that so that the false events are almost equally divided across it, as illustrated in Table 7. This results in balanced PPV and NPV values. At relatively higher epoch values, for getting deep learning features, the NPV is higher in comparison to the NPV without RA because of a relatively lower number of FNs (Table 7).

**Table 7:** Proposed system using original and modified datasets (with RA) (tenfold-cross validated) with epochs variations used for bleeding deep learning features depicting TP, TN, FP and FN counts *k*-NN classifier with *k* = 1 for BraTS-2021 dataset (with RA) based on deep learning features.

Epoch	Original dataset				Modified dataset with RA			
	TP	FN	TN	FP	TP	FN	TN	FP
1	29,521 ± 44	1049 ± 44	27,504 ± 3	11,654 ± 3	21,331 ± 41	1061 ± 41	27,396 ± 45	990 ± 45
5	29,640 ± 12	930 ± 3	27,597 ± 3	11,561 ± 5	21,465 ± 31	927 ± 31	27,558 ± 29	828 ± 29
10	29,499 ± 17	1071 ± 8	27,474 ± 7	11,684 ± 8	21,276 ± 00	1116 ± 00	27,485 ± 00	901 ± 00
15	29,166 ± 31	1403 ± 31	27,158 ± 60	11,999 ± 60	21,035 ± 33	1357 ± 33	27,168 ± 89	1218 ± 89
20	28,982 ± 117	1588 ± 117	26,973 ± 255	12,185 ± 255	20,852 ± 117	1540 ± 117	27,019 ± 02	1367 ± 02
25	28,942 ± 109	1628 ± 109	26,964 ± 102	12,194 ± 102	20,820 ± 45	1571 ± 45	26,901 ± 63	1485 ± 63
30	28,808 ± 213	1762 ± 213	26,934 ± 126	12,224 ± 126	20,817 ± 14	1574 ± 14	26,834 ± 42	1552 ± 42
40	28,702 ± 91	1867 ± 91	26,854 ± 106	12,303 ± 106	20,655 ± 69	1737 ± 69	26,795 ± 137	1590 ± 137
50	28,730 ± 54	1839 ± 54	26,823 ± 20	12,334 ± 20	20,709 ± 129	1682 ± 129	26,737 ± 150	1649 ± 150

#### SVM model

Three important variants of SVM classifier using linear, polynomial kernels of order ∈ (2,3,4) and radial basis function (RBF) have been experimented with to classify HFS into MGMT+ and MGMT− classes using latent features. The polynomials of higher-order (> 4) were not able to generate significant results. The results using SVM for binary classification of BraTS-2021 with RA using latent features extracted at the fifth epoch have been illustrated in Table 8. It can be observed that SVM RBF has achieved outclass performance among other variants for the proposed model.

**Table 8:** Proposed system results (tenfold-cross validated) with five epochs depicting analysis based on performance using SVM for BraTS-2021 dataset (with RA).

Classifier	S_p_ (%)	R_c_ (or S_n_) (%)	P_r_ (or PPV) (%)	NPV(%)	AUC (ROC)	F_1_-score	MCC	A (%)
SVM Linear	79.43 ± 0.03	41.70 ± 0.04	61.53 ± 0.01	63.33 ± 0.05	0.61 ± 0.03	0.49 ± 0.03	0.23 ± 0.03	62.79 ± 0.02
**SVM RBF**	**92.88 ± 0.03**	**82.84 ± 0.04**	**90.18 ± 0.04**	**87.12 ± 0.02**	**0.87 ± 0.02**	**0.86 ± 0.03**	**0.76 ± 0.02**	**88.46 ± 0.03**
SVM Poly 2	85.89 ± 0.02	89.83 ± 0.04	83.39 ± 0.03	91.46 ± 0.02	0.87 ± 0.01	0.86 ± 0.03	0.75 ± 0.03	87.63 ± 0.03
SVM Poly 3	85.38 ± 0.01	81.27 ± 0.03	81.43 ± 0.01	85.25 ± 0.03	0.83 ± 0.03	0.81 ± 0.01	0.66 ± 0.02	83.57 ± 0.01
SVM Poly 4	55.96 ± 0.02	84.99 ± 0.05	60.35 ± 0.03	82.05 ± 0.03	0.70 ± 0.01	0.70 ± 0.03	0.42 ± 0.01	68.76 ± 0.02

Significant values are given in bold.

Another study has been carried out by changing the number of folds in the range [10, 20,…,50] during cross-validation for both *k*-NN and SVM classifiers for accuracy and F_1_-score. The performance variation between different folds has been found insignificant and negligible. However, the time for classification rises with an increase in the number of folds. The optimum number of folds was found ten and used throughout the article.

### Performance analysis of DL-based latent feature extraction

The DLRFE module extracts the dynamic features from fully connected layers using the mpMRI scans. The convolution process is based on local operators, in case the kernel size is small, that can be thought of as a subset of fully connected layers, and the same is true for pooling, whereas the fully connected layers have a global concept so that each neuron is connected with every neuron in the next layer causing information to pass through each input to each output class. The final decision is based on the whole image^89^. The features in the first two fully connected layers, FC1 and FC2, are bled from the tuned architecture in the forward direction for the training and test partitions. The features are blended to form the latent features ${x}_{latent}$. The weight distribution in two fully connected layers is used to decide for either of the classes. The variation of the number of unknowns with a varying number of DL layers for the deep learning architecture is illustrated in Table 9 The parametric set finalized after empirical tuning is given in Table 10.

**Table 9:** Detail of learnable parameters with DL architecture layers.

Layer no	Name	Type	Activations	Learnables
1	**ImageInputLayer** 64 × 64x1 images with 'zerocenter' normalization	Image input	64 × 64 × 1	0
2,3	**Conv-1** 64 3 × 3 convolutions with stride [1 1] and padding 'same' + **ReLU-1**	Convolution, ReLU	64 × 64 × 64	Weights 3 × 3 × 1 × 64
				Bias 1 × 1 × 64
4	**MaxPool_1** 2 × 2 max pooling with stride [2 2] and padding [0 0 0 0]	Max pooling	32 × 32 × 64	0
5,6	**Conv-2** 128 3 × 3 convolutions with stride [1 1] and padding 'same' + **ReLU-2**	Convolution, ReLU	32 × 32 × 128	Weights 3 × 3 × 64 × 128
				Bias 1 × 1 × 128
7	**MaxPool_2** 2 × 2 max pooling with stride [2 2] and padding [0 0 0 0]	Max pooling	16 × 16 × 128	0
8	**BN-2**	Batch normalization	16 × 16 × 128	Offset 1 × 1 × 128
				Scale 1 × 1 × 128
9,10	**Conv-3** 256 3 × 3 convolutions with stride [1 1] and padding 'same' + **ReLU-3**	Convolution, ReLU	14 × 14 × 256	Weights 3 × 3 × 128 × 256
				Bias 512 × 1 × 256
11	**MaxPool_3** 2 × 2 max pooling with stride [2 2] and padding [0 0 0 0]	Max pooling	7 × 7 × 256	0
12	**Drop-1** 20% dropout	Dropout	7 × 7 × 256	0
13	**FC1** (512 neurons fully connected layer)	Fully connected	1 × 1 × 512	Weights 512 × 12,544
				Bias 512 × 1
14	**FC2** (64 neurons fully connected layer)	Fully connected	1 × 1 × 64	Weights 64 × 512
				Bias 64 × 1
15	**FC3** (2 neurons fully connected layer)	Fully connected	1 × 1 × 2	Weights 2 × 64
				Bias 2 × 1
17	**SM**	Softmax	1 × 1 × 2	0
18	**Class**	Classification output	1 × 1 × 2	0

**Table 10:** Deep learning parameters used for latent features extraction using DLRFE module.

DL parameters	Optimal values
Solver name	SGDM
Momentum (sgdm)	0.99
Initial learning rate	Single: 0.0001
Epochs (maximum)	50
Mini batch (size)	16
Pairs of Conv. layers and filter stacks	3
Kernel depth (size of each filter stack)	64, 128, 256
Kernel size	3 × 3
ReLU layers	3
Pooling type	MaxPool (2 × 2)
Fully connected layers for latent features	2
Number of filter stacks	3
L2-regularization	0.0001
Input image size	64 × 64
Loss function	Cross entropy
Number of dropout layers	1
Dropout rate (%)	20

### Analysis of parameters for deep learning and radiomic feature methods

The deep learning (latent) and radiomic feature extraction, constituting stages 1 and 2 of the proposed methodology, involves several parameters that need to be addressed by selecting their optimum values before the formation of HFS. We address the selection of appropriate values of parameters by an experimental process for each of the categories of feature extraction in a sequential manner.

#### Feature space visualization of deep learning-based FC1 and FC2

**Figure 11:**
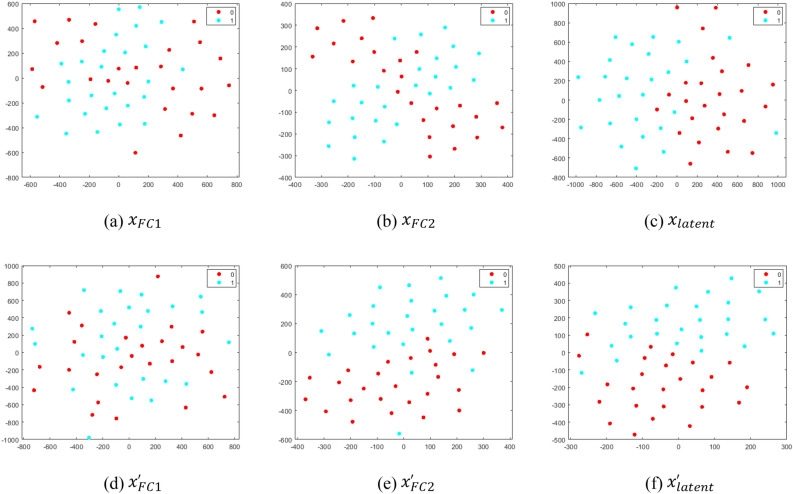
The scatter plot for latent features mapped to ℜ^2^ using the t-SNE technique illustrating Stage 1 for classification of mpMRI scans into MGMT+ (“1”) and MGMT− (“0”) classes for the original dataset: (**a**) FC1 features ${x}_{FC1}$, (**b**) FC2 features ${x}_{FC2}$, and (**c**) latent features ${x}_{latent}$. The results depicting visual classification with the rejection algorithm are: (**d**) ${x}_{FC1}^{^{\prime}}$ (**e**) ${x}_{FC2}^{^{\prime}}$, and (**f**) ${x}_{latent}^{^{\prime}}$.

The t-SNE plots, a variant of Stochastic Neighborhood Embedding (SNE), for radiogenomic classification for the test cases using deep learning-based features, namely $${x}_{FC1}$$, $${x}_{FC2}$$, and $${x}_{latent}$$, are illustrated in Fig. 11a–c, d–f using the 1-NN classifier ($$k=1)$$, "Performance analysis of DL-based latent feature extraction", with original dataset and reduced dataset with RA applied respectively. For each of the high-dimensional feature vectors, the mapping is carried out in a non-linear manner to a lower (two) dimensional plan. The improvement in visualization is attributed solely to the alleviation of the tendency of points-crowding in the central region. Although there are marked inclinations of false events in FC1 and FC2 without RA due to overlapping regions, Fig. 11a–c, the fused DL features improved the results. Similarly, as illustrated in Fig. 11d–f using RA, the features represented the discrimination of vectors lying in either of the classes. Further improvement, by the exploitation of features, is carried out by our proposed system with the notion to find the complex hyperplane causing discrimination between MGMT+ and MGMT− classes.

#### GLCM feature extraction process

**Figure 12:**
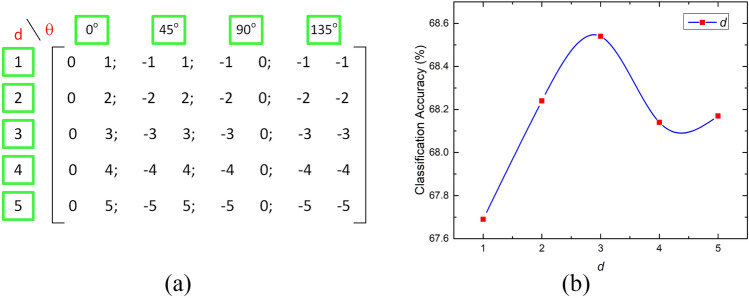
The variation of classification performance accuracy using GLCM feature descriptor for different values of *d* with variation in *θ* as 0°, 45°, 90°, and 135°, (**a**) Offset matrix, and (**b**) accuracy versus *d* plot.

We experimented with five distance values in the range^1,5^, each corresponding to a GLCM with four directions of *θ,* and each value of GLCM corresponds to its directional average corresponding to a specific value of *d*. The independent values of *θ*, without averaging, resulted in four GLCMs, each corresponding to a specific direction. So, a total of 20 GLCMs were generated without direction averaging. Further, several combinations of GLCMs and the hybrids of features thereof, based on cross-averaging of GLCMs using different combinations of *θ*, were also investigated for the classification of mpMRI scans to MGMT+ and MGMT− classes. The performance of GLCM features varies with the value of *d*. The array of offset matrices used for this experimentation to try different combinations of *d* and *θ* has been shown in Fig. 12a. The perception of the relationship between *d* and accuracy is illustrated in Fig. 12b. Further, the best results for our problem were found at ($d=3$) with direction averaging of four angles incremented by 45° without the repeated symmetric view. Initially, the classification accuracy shows a rising trend, and finally, accuracy falls off with a local rising surge. But the peak value of accuracy has been found at $d=3$, therefore, this value of the relative distance from the neighboring pixels has been used for the feature extraction process.

#### HOG feature extraction process

**Figure 13:**
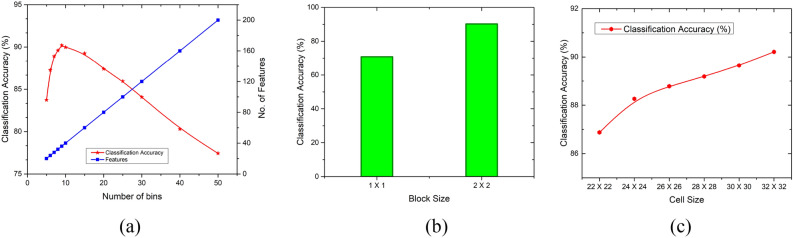
Sensitivity analysis of HOG parameters for radiogenomic classification: (**a**) number of bins, (**b**) block size, and (**c**) cell size.

The investigation was carried out to tune the parameters of the feature descriptor for our specific problem of radiogenomic classification. Further, we tried individual HOG parameters, based on classification accuracy, between the number of bins, block- and cell-sizes as illustrated in Fig. 13. Here, Fig. 13a shows the trend for the effects of varying the number of bins. Initially, the classification accuracy shows a rising trend, and finally, accuracy falls off. But the peak value of accuracy has been found at the uphill of the initial part. There is a sort of compromise found between the number of features and generalization. The higher number of features, due to the higher number of bins, resulted in dropped performance due to the curse of dimensionality. Figure 13b shows the effect of block size on possible options depending on the input image size. The higher block size resulted in outclass performance with the number of bins selected as 9 and block size as 32 × 32. Figure 13c shows the plot for cell size variation with classification accuracy. Higher cell size, before the maximum possible value, resulted in increased classification performance.

The features exploiting the large-scale spatial characteristics are based on a large HOG cell size. Consequently, the small-scale related features are lost with large cell sizes. Therefore, a compromise exists between the cell size and the details required depending on the inherent structure of the problem being experimented with. Similarly, local illumination changes are explored at a smaller HOG block size as the important information is lost when averaging the pixel intensities in a large block (with a relatively larger number of pixels). Consequently, the significant changes in the local pixels can be explored by reducing the block size. The parameters that play a key role in attaining its best individual performance were determined using the *k*-NN classifier, and the optimum selected values are illustrated in Table 11 out of the under-trial options. The optimum results for our problem structure were found using cell size: 32 × 32, and block size: 2 × 2 based on 9 bins resulting in ${x}_{HOG}=$ 36.

**Table 11:** Optimal parameters of HOG feature extraction module.

Parameter	Options (valid under trial)	Number of features	Optimum selection
Number of bins	5, 6, 7, 8, 9, 10, 15, 20, 25, 30, 40, 50	20, 24, 28, 32, 36, 40, 60, 80, 100, 120, 160, 200	9
Block size	1 × 1, 2 × 2	36 (each option)	2 × 2
Cell size	22 × 22, 24 × 24, 26 × 26, 28 × 28, 30 × 30, 32 × 32	36 (each option)	32 × 32

#### LBP features

**Figure 14:**
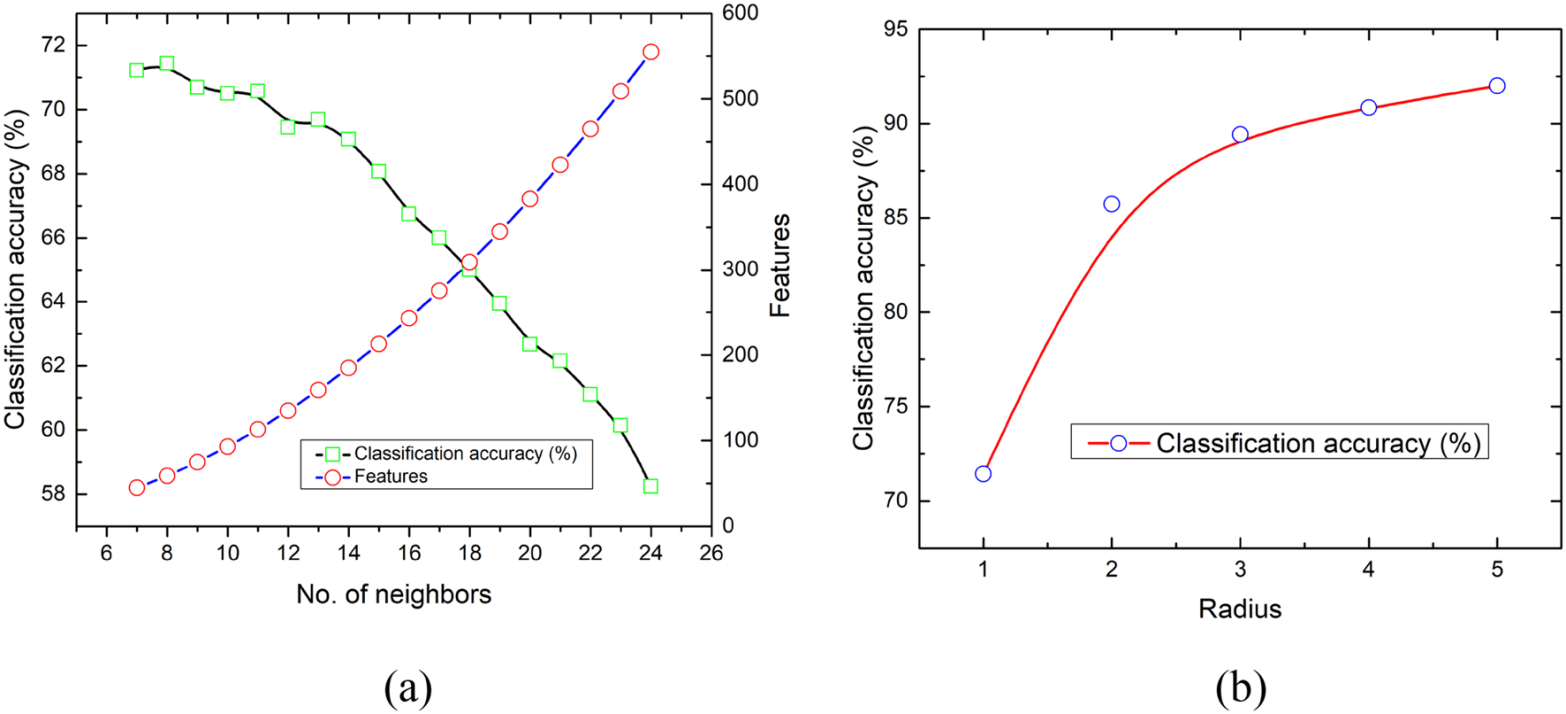
The sensitivity analysis of LBP parameters: (**a**) the number of neighbors, and (**b**) the radius.

We investigated different parameters of the LBP feature extraction module that significantly affected its performance. The trials were based on the number of neighbors *p*, radius *r*, and the receptive area to get an insight into the relationship between these parameters and the model performance for radiogenomic classification. Figure 14 shows the effect of individual LBP parameters on radiogenomic classification. Figure 14a shows the trend of classification accuracy with *p* so that increasing the latter encodes more details corresponding to the surroundings of each pixel. The optimum classification accuracy is 70.44% associated with a minimum number of features as 59 and $r=1$. Similarly, Fig. 14b illustrates how varying *r*, the pointing boundary for the circular pattern through which neighbors are selected, influences the performance in terms of classification accuracy. The accuracy is 92.11% using the optimum value of *p* for $r=5$. The optimal parameters for the LBP feature extraction module are illustrated in Table 12. The experimentation for the LBP parametric analysis was carried out using 1-NN classifier with RA that significantly influenced the performance of the framework.

**Table 12:** Optimal parameters of LBP feature extraction module with cell size as 64 × 64.

Parameter	Options (valid under trial)	Number of features	Optimum selection
Number of neighbors	7, 8, 9, 10, 11, 12, 13, 14, 15, 16, 17, 18, 19, 20, 21, 22, 23, 24	45, 59, 75, 93, 113, 135	8 (59)
Radius	1–5	59 (each option)	5

### Performance analysis of individual FEMs

**Figure 15:**
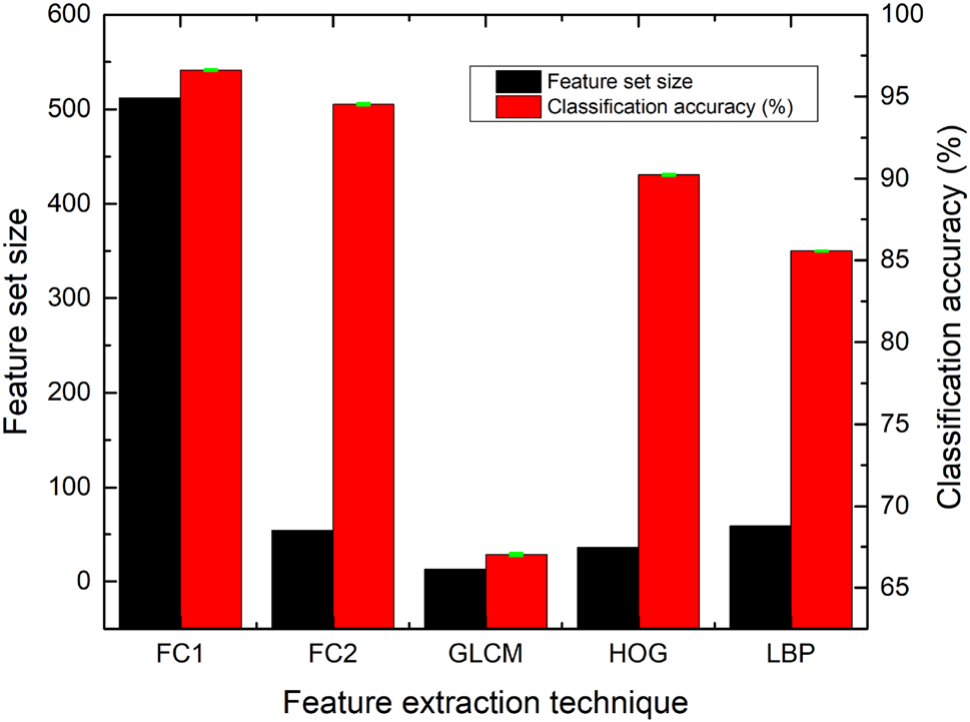
Comparison of feature extraction techniques for their feature set size and the resulting classification accuracy with RA using a $1$-NN classifier.

The various *k*-values for *k*-NN along with important SVM variants have been tried but 1-NN has been found outclass. The various performance measures have been illustrated in Table 13 illustrating the corresponding results using BraTS-2021 with and without RA. Each of the extraction modules has been found to accompany reasonable performance measuring indices. However, a close analysis of the results reveals that the proposed latent features with RA perform relatively better comparing all the performance metrics. Similarly, HOG features, based on the structure of the problem with RA, have also accompanied adequate classification results. Figure 15 shows a comparison of various feature sets for their size and classification performance accuracy. The FC2, using the proposed deep learning architecture, is claimed to have a marked effect in comparison to its share (9.35%) in the HFS. The next optimum in this respect is the HOG set, with optimized parameters for our problem structure, with a 5.26% share in the HFS resulting in remarkable individual accuracy.

**Table 13:** RA effect on Radiogenomic classification results for individual feature extraction modules using a 1-NN classifier and BraTS-2021 dataset.

Features	RA	S_p_ (%)	R_c_ (or S_n_) (%)	P_r_ (or PPV) (%)	NPV (%)	AUC (ROC)	F1-score	MCC	A (%)
FC1	**√**	96.98 ± 0.03	96.13 ± 0.02	96.17 ± 0.04	96.95 ± 0.05	0.97 ± 0.03	0.96 ± 0.02	0.93 ± 0.01	96.61 ± 0.03
FC2		95.23 ± 0.04	93.65 ± 0.02	93.94 ± 0.01	95.01 ± 0.04	0.94 ± 0.04	0.94 ± 0.03	0.89 ± 0.02	94.54 ± 0.04
GLCM		70.89 ± 0.18	62.16 ± 0.07	62.75 ± 0.12	70.37 ± 0.04	0.67 ± 0.01	0.62 ± 0.08	0.33 ± 0.02	67.04 ± 0.08
HOG		91.57 ± 0.03	88.53 ± 0.05	89.23 ± 0.05	91.01 ± 0.04	0.90 ± 0.01	0.89 ± 0.05	0.80 ± 0.01	90.23 ± 0.04
LBP		87.70 ± 0.16	83.52 ± 0.13	84.27 ± 0.11	87.09 ± 0.10	0.86 ± 0.02	0.84 ± 0.12	0.71 ± 0.01	85.56 ± 0.01
FC1	×	70.57 ± 0.04	97.01 ± 0.02	72.02 ± 0.08	97.12 ± 0.05	0.84 ± 0.02	0.83 ± 0.03	0.68 ± 0.02	82.16 ± 0.03
FC2		69.26 ± 0.02	95.42 ± 0.03	70.79 ± 0.04	95.32 ± 0.06	0.82 ± 0.04	0.81 ± 0.02	0.65 ± 0.01	80.73 ± 0.04
GLCM		79.15 ± 0.15	45.58 ± 0.08	63.06 ± 0.11	65.88 ± 0.03	0.62 ± 0.02	0.52 ± 0.09	0.26 ± 0.03	64.43 ± 0.08
HOG		66.16 ± 0.04	91.19 ± 0.05	67.78 ± 0.04	91.90 ± 0.04	0.78 ± 0.02	0.77 ± 0.04	0.57 ± 0.02	77.13 ± 0.05
LBP		63.06 ± 0.10	86.96 ± 0.11	64.76 ± 0.09	86.36 ± 0.08	0.75 ± 0.03	0.74 ± 0.10	0.50 ± 0.02	73.54 ± 0.02

### Performance analysis of HFS

**Figure 16:**
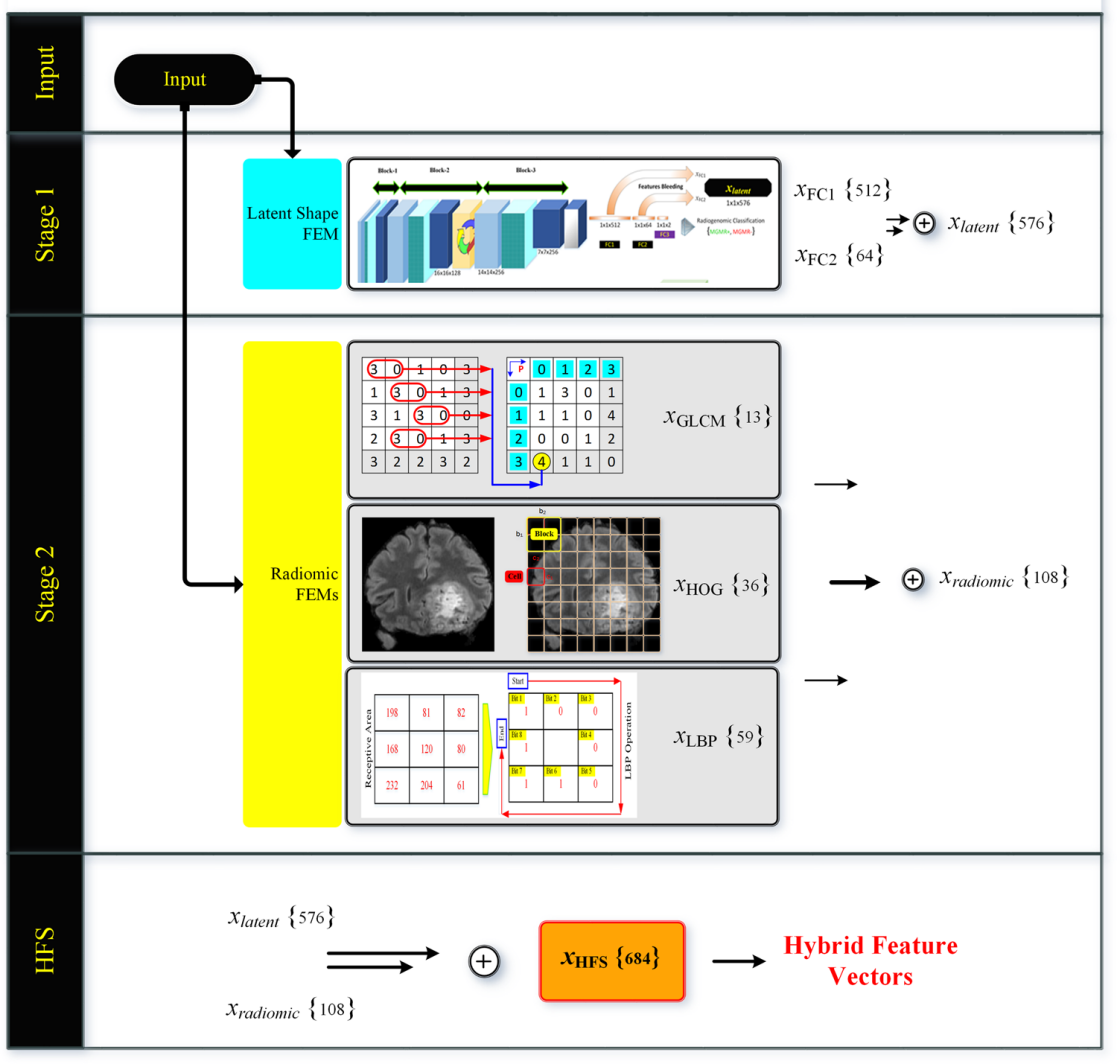
Schematic diagram of multi-omics HFS formation for the proposed classification framework.

This set of experiments is based on a combinatorial strategy using individual features from both stages, i.e. latent features from Stage 1 and radiomic features from Stage 2. The distribution of features in HFS into various categories and their proportionate share is illustrated in Table 14. The 2-Stage HFS formation for the proposed radiogenomic classification framework is illustrated in Fig. 16. The feature extraction modules are fused in this trial by manifold combinations, each consisting of two, three, four, and five feature types. Each of the experiments is evaluated using BraTS-2021 with RA using various performance measuring techniques as illustrated in Tables 15, 16 and 17. FC1, FC2, and HOG features resulted in an adequate level of performance after the application of RA on the dataset no matter whether they are utilized on an individual basis or in cross-breed mode. Results in Tables 13 and 15 concluded that individual features, FC1, FC2, and HOG, yielded maximum accuracy of 96.61%, 94.54%, and 90.23% respectively whereas the accuracy was further boosted with an increase up to 96.88% in the case of hybrid of more than two features. In Tables 16 and 17, combinations of three (10 sets), four (5 sets), and five features (1 set) were hybridized to form HFS using a *k*-NN classifier with RA to yield a maximum accuracy of 96.94%. However, a slight fractional enhancement in performance resulted in complex hybrids carrying more than two feature subsets, leading to the accuracy up to 96.90%, 96.92%, and 96.94% for FC1-GLCM-HOG, FC1-GLCM-HOG-LBP, and FC1-FC2-GLCM-HOG-LBP (HFS) respectively. The outclass results are mainly due to the reduction of similar features in the original dataset by the application of RA so that the diversity in HFS is combined by exploiting five distinct feature extraction modules. Each of the extraction modules, having its possible solution domain, extracts the most favorable and discriminative characteristics from the mpMRI scans. When the features originated from the RA-based reduced BraTS-2021 dataset fused, they collectively generated better results, and an overall accuracy increase of 17.59% and 17.99% was observed for individual features’ performance and hybrid strategies respectively when compared with results without RA.

**Table 14:** Features forming HFS for radiogenomic classification using the BraTS-2021 dataset.

Definition (features)	Source	Share (%)	Count (features)
*x*_FC1_	DL module, fully connected layer 1(512 neurons)	74.85	512
*x*_FC2_	DL module, fully connected layer 2(64 neurons)	9.35	64
*x*_latent_	Entire DL feature contribution	84.21	576
*x*_GLCM_	GLCM based direction-averaged features	1.90	13
*x*_HOG_	HOG based	5.26	36
*x*_LBP_	LBP based	8.62	59
*x*_radiomic_	Radiomic feature contribution	15.79	108
*x*_HFS_	Hybrid features set	100.00	684

**Table 15:** Sensitivity analysis manifold combinations of two features using a 1-NN classifier and BraTS-2021 dataset with RA.

Features	S_p_ (%)	R_c_ (or S_n_) (%)	P_r_ (or PPV) (%)	NPV (%)	AUC (ROC )	F1-Score	MCC	A (%)
FC1-FC2	97.08 ± 0.10	95.86 ± 0.14	96.29 ± 0.13	96.80 ± 0.13	0.96 ± 0.05	0.96 ± 0.13	0.93 ± 0.03	96.54 ± 0.10
FC1-GLCM	97.02 ± 0.04	96.01 ± 0.05	96.21 ± 0.02	96.82 ± 0.02	0.96 ± 0.02	0.96 ± 0.04	0.93 ± 0.01	96.57 ± 0.04
**FC1-HOG**	**97.24 ± 0.03**	**96.44 ± 0.05**	**96.50 ± 0.03**	**97.23 ± 0.01**	**0.96 ± 0.03**	**0.96 ± 0.04**	**0.93 ± 0.01**	**96.88 ± 0.03**
FC1-LBP	97.10 ± 0.02	96.23 ± 0.04	96.32 ± 0.03	97.02 ± 0.01	0.97 ± 0.01	0.96 ± 0.03	0.93 ± 0.01	96.71 ± 0.02
FC2-GLCM	95.32 ± 0.01	93.51 ± 0.02	94.03 ± 0.03	94.91 ± 0.02	0.94 ± 0.02	0.93 ± 0.01	0.89 ± 0.02	94.52 ± 0.01
FC2-HOG	96.19 ± 0.02	94.84 ± 0.03	95.16 ± 0.01	95.94 ± 0.02	0.95 ± 0.03	0.95 ± 0.02	0.91 ± 0.02	95.59 ± 0.02
FC2-LBP	95.55 ± 0.01	93.94 ± 0.01	94.34 ± 0.02	95.24 ± 0.03	0.94 ± 0.01	0.94 ± 0.01	0.89 ± 0.02	94.84 ± 0.01
GLCM-HOG	93.69 ± 0.14	91.00 ± 0.30	91.92 ± 0.17	92.96 ± 0.22	0.92 ± 0.00	0.91 ± 0.00	0.85 ± 0.02	92.51 ± 0.16
GLCM-LBP	88.43 ± 0.11	83.98 ± 0.34	85.13 ± 0.14	87.50 ± 0.24	0.86 ± 0.01	0.85 ± 0.01	0.73 ± 0.00	86.47 ± 0.17
HOG-LBP	91.74 ± 0.14	88.88 ± 0.16	89.46 ± 0.17	91.27 ± 0.12	0.90 ± 0.01	0.89 ± 0.00	0.81 ± 0.01	90.48 ± 0.14

Significant values are given in bold.

**Table 16:** Sensitivity analysis manifold combinations of three features using a 1-NN classifier and BraTS-2021 dataset with RA.

Features	S_p_ (%)	R_c_ (or S_n_) (%)	P_r_ (or PPV) (%)	NPV (%)	AUC (ROC)	F1-score	MCC	A (%)
FC1-FC2-GLCM	97.01 ± 0.01	95.86 ± 0.02	96.19 ± 0.02	96.74 ± 0.01	0.96 ± 0.01	0.96 ± 0.01	0.92 ± 0.01	96.50 ± 0.02
FC1-FC2-HOG	97.22 ± 0.02	96.27 ± 0.01	96.47 ± 0.01	97.07 ± 0.01	0.96 ± 0.01	0.96 ± 0.01	0.93 ± 0.01	96.80 ± 0.01
FC1-FC2-LBP	97.05 ± 0.03	96.16 ± 0.01	96.26 ± 0.02	96.97 ± 0.02	0.96 ± 0.02	0.96 ± 0.01	0.93 ± 0.02	96.66 ± 0.02
FC2-GLCM-HOG	96.58 ± 0.02	95.05 ± 0.01	95.64 ± 0.03	96.11 ± 0.02	0.95 ± 0.01	0.95 ± 0.01	0.91 ± 0.02	95.91 ± 0.01
FC2-GLCM-LBP	95.50 ± 0.03	93.73 ± 0.02	94.26 ± 0.01	95.07 ± 0.03	0.94 ± 0.02	0.94 ± 0.02	0.89 ± 0.03	94.72 ± 0.04
FC2-HOG-LBP	96.09 ± 0.01	94.87 ± 0.02	95.03 ± 0.01	96.06 ± 0.01	0.95 ± 0.04	0.95 ± 0.01	0.91 ± 0.02	95.55 ± 0.03
FC1-GLCM-HOG	**97.32 ± 0.01**	**96.27 ± 0.01**	**96.60 ± 0.02**	**97.06 ± 0.02**	**0.96 ± 0.03**	**0.96 ± 0.01**	**0.93 ± 0.01**	**96.90 ± 0.01**
FC1-GLCM-LBP	97.09 ± 0.02	96.13 ± 0.02	96.31 ± 0.01	96.95 ± 0.02	0.96 ± 0.02	0.96 ± 0.02	0.93 ± 0.02	96.67 ± 0.04
FC1-HOG-LBP	63.92 ± 0.04	40.15 ± 0.05	46.75 ± 0.03	57.52 ± 0.02	0.52 ± 0.01	0.43 ± 0.04	0.041 ± 0.04	53.44 ± 0.04
GLCM-HOG-LBP	92.52 ± 0.01	89.17 ± 0.02	90.39 ± 0.01	91.54 ± 0.02	0.90 ± 0.03	0.89 ± 0.04	0.81 ± 0.03	91.04 ± 0.01

Significant values are given in bold.

**Table 17:** Sensitivity analysis manifold combinations of four and five features using a 1-NN classifier and BraTS-2021 dataset with RA.

Features	S_p_ (%)	R_c_ (or S_n_) (%)	P_r_ (or PPV) (%)	NPV (%)	AUC (ROC)	F1-score	MCC	A(ML) (%)
FC1-FC2-GLCM-HOG	97.25 ± 0.02	96.16 ± 0.01	96.51 ± 0.01	97.01 ± 0.01	0.96 ± 0.01	0.96 ± 0.01	0.93 ± 0.01	96.77 ± 0.01
FC1-FC2-HOG-LBP	97.30 ± 0.02	96.40 ± 0.02	96.57 ± 0.02	97.10 ± 0.02	0.96 ± 0.01	0.96 ± 0.02	0.93 ± 0.02	96.90 ± 0.02
FC1-FC2-GLCM-LBP	97.05 ± 0.01	96.07 ± 0.02	96.26 ± 0.02	96.90 ± 0.02	0.96 ± 0.01	0.96 ± 0.01	0.93 ± 0.02	96.62 ± 0.01
FC2-GLCM-HOG-LBP	96.21 ± 0.03	94.83 ± 0.04	95.18 ± 0.01	95.94 ± 0.01	0.95 ± 0.02	0.95 ± 0.03	0.91 ± 0.01	95.60 ± 0.01
**FC1-GLCM-HOG-LBP**	**97.42 ± 0.01**	**96.29 ± 0.01**	**96.71 ± 0.01**	**97.09 ± 0.02**	**0.96 ± 0.01**	**0.96 ± 0.01**	**0.93 ± 0.02**	**96.92 ± 0.02**
**HFS**	**97.44 ± 0.01**	**96.31 ± 0.03**	**96.60 ± 0.01**	**97.10 ± 0.02**	**0.96 ± 0.01**	**0.96 ± 0.01**	**0.94 ± 0.02**	**96.94 ± 0.01**

Significant values are given in bold.

From another perspective of sensitivity analysis of epochs on proposed deep learning features extraction, and consequently on ML-based classification has been illustrated in Table 18 on the radiongenomic classification dataset with RA. The epochs when larger than five marginally influence, 0.8% increase, the performance measuring standards with radiomic as static feature extraction techniques.

**Table 18:** Proposed MGMT-PMP system results cross-validated 10-folds with epochs variation for dynamic feature extraction depicting analysis based on performance with RA, using 1-NN classifier for BraTS-2021 dataset.

Epoch	S_p_ (%)	R_c_ (or S_n_) (%)	P_r_ (or PPV) (%)	NPV (%)	AUC (ROC)	F_1_-score	MCC	A (%)
1	97.14 ± 0.07	95.78 ± 0.16	96.36 ± 0.09	96.69 ± 0.12	0.96 ± 0.00	0.96 ± 0.00	0.93 ± 0.00	96.54 ± 0.09
**5**	**97.43 ± 0.01**	**96.31 ± 0.03**	**96.60 ± 0.01**	**97.10 ± 0.02**	**0.96 ± 0.01**	**0.96 ± 0.01**	**0.94 ± 0.02**	**96.93 ± 0.01**
10	97.20 ± 0.24	96.06 ± 0.24	96.44 ± 0.29	96.90 ± 0.18	0.97 ± 0.00	0.96 ± 0.00	0.93 ± 0.00	96.70 ± 0.14
15	96.83 ± 0.18	95.17 ± 0.28	95.95 ± 0.23	96.21 ± 0.22	0.96 ± 0.00	0.96 ± 0.00	0.92 ± 0.00	96.10 ± 0.23

Significant values are given in bold.

### Computational time analysis of the framework

It is worthwhile to analyze the proposed framework for its space and time complexity in terms of CPU time requirements. To check the performance of the framework, the CPU time involvement has been considered for various feature extraction modules on an individual as well as group basis, using ML/ DL classifiers after applying RA, and training/ testing time on a per-image basis. The hybrid feature extraction time is the mere sum of the time taken by the individual FEMs.

**Figure 17:**
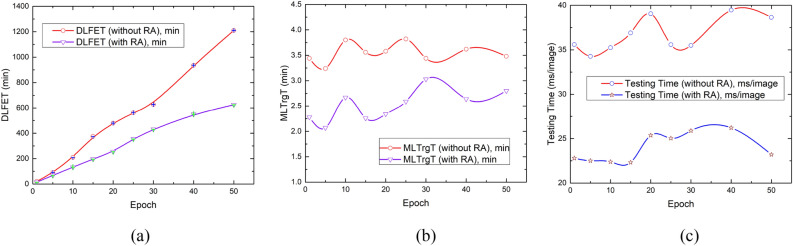
Effect of RA on time required for (**a**) deep learning feature extraction time (minutes), (**b**) ML training time and (**c**) ML testing time for radiogenomic classification using BraTS-2021 dataset and 1-NN classifier (DLFET stands for deep learning feature extraction time and MLTrgT stands for Machine Learning Training Time).

The time requirements (with epochs) for deep learning feature extraction in minutes, machine learning training time (MLTrgT) in minutes, and machine learning testing time (MLTstT) in ms/ image are illustrated in Fig. 17a–c as DLFET, MLTrgT and MLTstT respectively. Figure 17a shows that the latent feature extraction time rises with epochs, and five epochs have been found optimum ("Selection of optimal parameters") so that the HFS culminates in high classification performance accuracy. Further, RA application resulted in a reduction of feature extraction time, so there is a time gap observed between the two trends.

In Fig. 17b, the training time for machine learning remained almost uniform. The variation on the *x*-axis represents the epochs used at which the latent features were extracted. Further, the training features are obtained by finding activations through deep learning architecture in the forward direction. The latent features are then used by a 1-NN classifier for model training. The curve related to the RA application used lesser time in comparison to the original dataset having a larger number of training instances. A similar trend has been shown in Fig. 17c in milliseconds for testing on a per-image basis using a 1-NN classifier. The testing time on a per-image curve is always accompanied by quick convergence in the case of RA.

**Figure 18:**
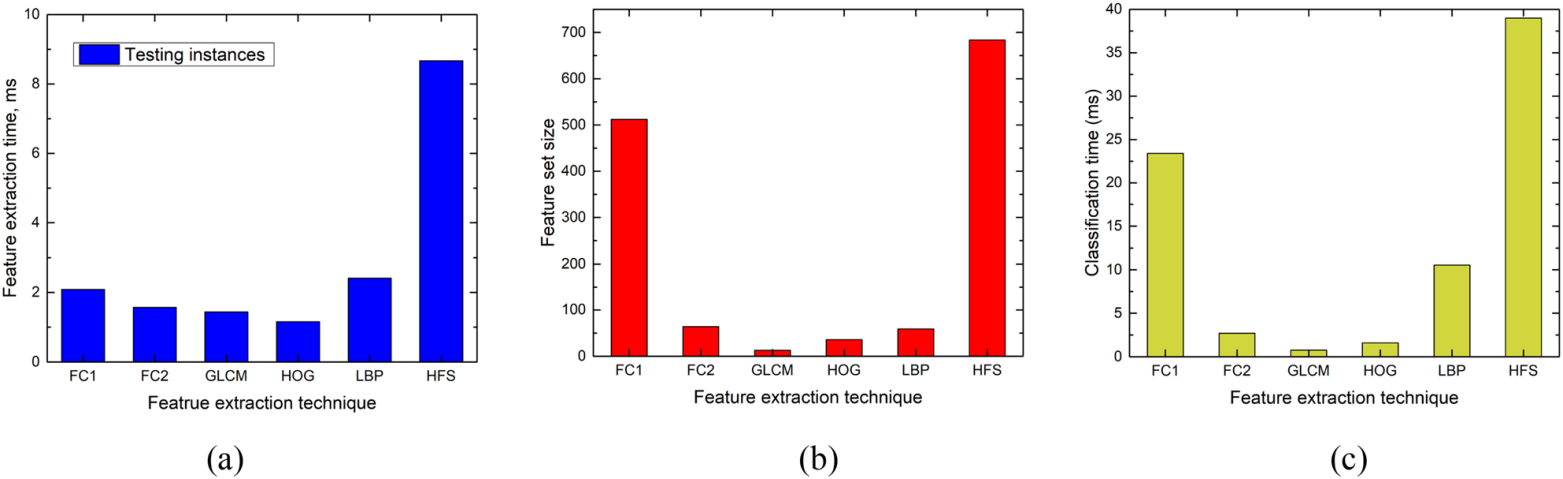
Comparison of (**a**) feature extraction times for potential feature extraction techniques with (**b**) individual feature set size used in the proposed framework, and (**c**) classification time without preprocessing and feature extraction with RA using BraTS-2021 dataset and 1-NN classifier.

The trend of feature extraction time/image has been demonstrated in Fig. 18a. Although a relatively higher extraction cost for FET is observed for LBP, attributed to its large radius that was found optimum considering its contribution on an individual basis ("LBP features"), the proposed system is tractable computationally, only 8.67 ms for the HFS extraction, due to time involvement per feature extraction module. However, the preprocessing times, the training time for deep features extraction, and the RA times are not included in these computations as they are to be involved once, and from thereon never to be repeated, until and unless new test data is involved which has to follow the same norms as defined earlier. Further, the higher time cost of LBP is justifiable by performance rise compared to other FEMs. Figure 18b illustrates the feature vector size for individual and hybrid features, where the latter is just the summation of the former. Figure 18c shows the classification time for individual FEMs and their hybrid. The classification time varies with the length of the feature vector, and the classification time of HFS is high compared to the individual FEMs.

### Performance comparison

The classification performance of our proposed framework has been related to existing solutions for brain tumor classification using well-known radiomic features through the CE-MRI dataset. The BraTS-2021 dataset, based on radiogenomic data, has no published work so far to the best of our knowledge. In this context, we have implemented four techniques^17,40,79,90^ from the contemporary literature with optimal parameters for our comparison with the BraTS-2021 dataset. We used accuracy as the comparative performance measure. The tabular comparison has been illustrated in Table 19, which shows relatively better performance of the proposed framework over others in terms of performance. The radiomic features are texture oriented while latent features are problem structure oriented. The proposed system, however, uses discriminative HFS feature vectors wherein each module acquires knowledge associated with the problem structure. The manifold combinatorial fusion of different modules reinforces the multi-solution domain culminating in relatively superior performance measures.

**Table 19:** Comparison of the proposed framework with other studies for brain tumors using the BraTS-2021 challenge dataset using 80:20 partition for training: testing instances.

References	RA	Epochs	Technique	F_1_-score	A (%)
Qureshi^40^	×	20	UL-DLA + GLCM + SVM	0.74	75.24
Sultan^17^	×	20	CNN	0.67	68.42
Kaplan^79^	×	20	*n*LBP + *k*-NN	0.73	71.55
Badza^90^	×	20	CNN	0.71	73.55
Proposed framework (without RA)	×	5	MGMT-PMP system	0.83	83.16
**Proposed framework**	**√**	**5**	**MGMT-PMP system + RA**	**0.96**	**96.94**

Significant values are given in bold.

The effect of RA on radiogenomic classification accuracy is being reported with an improvement of 16.57% in the case using the reduced dataset exploited by RA. The outclass performance of the proposed MGMT-PMP system with RA is based on three key facts: firstly, simpler model, thereby reducing the complexity of the algorithm due to more focused/ highly discriminative features from the dataset using two-stage HFS, secondly, the computational cost is reduced due to the rejection of 27.18% instances, and thirdly, resulting in an improved generalization on testing instances with more discriminative feature vectors.

### Future challenges and recommendations

The main challenge to the proposed system is that it needs a clinical trial for resolving the expert opinion corresponding to the patient’s data with a second opinion. Another challenge is to define a systematic clinical step-up culminating in patient management with GB. Moreover, a study is required using the proposed technique for evaluation of its impact on the survival rate of GB patients with an associated clinical management system.

The deep learning-based prediction strategies, working as a source of latent feature extractor, are complex as well as opaque where performance metrics, viz. accuracy, F_1_-score, sensitivity, and specificity, are dependent on enormous parametric space using deep learning algorithms. In this context, the XAI^91,92^ proposed that the deep learning architectures should be examined using a white box that is transparent for multi-modal data fusion.

## Conclusions

In this research activity, we proposed a novel classification system MGMT-PMP, bridging imaging and genomics, for predicting radiogenomics classes using genetic variation associated with response to radiations. In this context, a novel fine-tuned CNN architecture, namely the DLRFE module is proposed for latent feature extraction to capture the features that dynamically bleed the quantitative knowledge related to the spatial distribution and the size of tumorous structure using the brain paraphernalia by mpMRI scans. It finally results in the development of a dynamic feature vector that is used in the radiogenomic classification. Further, in the proposed scheme, several radiomic features, namely GLCM, HOG, and LBP are extracted from mpMRI scans using the BraTS-2021 challenge dataset. The application of the novice rejection algorithm has been found very effective in selecting and delineating the instances out of the main dataset. The fusion of latent features with radiomic features, forming a hybrid feature collection as HFS, is then used in a different number of neighbors using *k*-NN classification. Working with mpMRI scans, using k-NN ($$k=1)$$ resulted in 97.28% training and 96.94% test classification accuracy. We have compared the proposed technique with numerous existing brain tumor classification techniques with the BraTS-2021 dataset and observed a significant increase in radiogenomic classification performance using RA. It has been established through results that dynamic- and static-feature fusion, using HFS, culminated in classification performance improvement as compared to individual features. This research study can have many implications extendable to multiple directions. First, this study links the genomic variation with radiomic-based data characterization algorithms paving the bridge between the two independent areas of research. The second possibility is its comprehensive use in real-time brain tumor surgery for the removal of leftover tumor cells by chemotherapeutic treatment as an alternative in tie to radiotherapy. Third, the proposed system can be used as an alternative without any dedicated machine as a portable low-cost solution for brain surgery. Further, this study can be of great impact on the clinical management and survival of GB patients.

## Data Availability

The use of all data mentioned in this article is publicly available at: https://www.kaggle.com/competitions/rsna-miccai-brain-tumor-radiogenomic-classification/data.
