# Author Correction: Radiogenomic classification for MGMT promoter methylation status using multi-omics fused feature space for least invasive diagnosis through mpMRI scans

**DOI:** 10.1038/s41598-023-32199-y

**Published:** 2023-03-30

**Authors:** Shahzad Ahmad Qureshi, Lal Hussain, Usama Ibrar, Eatedal Alabdulkreem, Mohamed K. Nour, Mohammed S. Alqahtani, Faisal Mohammed Nafie, Abdullah Mohamed, Gouse Pasha Mohammed, Tim Q. Duong

**Affiliations:** 1 Department of Computer and Information Sciences, Pakistan Institute of Engineering and Applied Sciences, Islamabad, Pakistan; 2 Department of Computer Science and IT, Neelum Campus, The University of Azad Jammu and Kashmir, Muzaffarabad, Azad Kashmir Pakistan; 3 Department of Computer Science and IT, King Abdullah Campus, The University of Azad Jammu and Kashmir, Muzaffarabad, Azad Kashmir Pakistan; 4 Department of Radiology, Albert Einstein College of Medicine and Montefiore Medical Center, 111 East 210th Street, Bronx, NY 10467 USA; 5 Farooq Hospital, Lahore, Pakistan; 6 Department of Computer Sciences, College of Computer and Information Sciences, Princess Nourah Bint Abdulrahman University, P.O. Box 84428, Riyadh, 11671 Saudi Arabia; 7 Department of Computer Sciences, College of Computing and Information System, Umm Al-Qura University, Mecca, Saudi Arabia; 8 Radiological Sciences Department, College of Applied Medical Sciences, King Khalid University, Abha, 61421 Saudi Arabia; 9 Department of Computer Science, College of Science and Humanities at Alghat, Majmaah University, Al-Majmaah, 11952 Saudi Arabia; 10 Research Centre, Future University in Egypt, New Cairo, 11845 Egypt; 11 Department of Computer and Self Development, Preparatory Year Deanship, Prince Sattam Bin Abdulaziz University, AlKharj, Saudi Arabia

Correction to: *Scientific Reports* https://doi.org/10.1038/s41598-023-30309-4, published online 25 February 2023

The Acknowledgements section in the original version of this Article was incomplete.

“Princess Nourah bint Abdulrahman University Researchers Supporting Project number (PNURSP2022R161), Princess Nourah bint Abdulrahman University, Riyadh, Saudi Arabia. The authors would like to thank the Deanship of Scientific Research at Umm Al-Qura University for supporting this work by Grant Code: (22UQU4310373DSR40).”

now reads:

“Princess Nourah bint Abdulrahman University Researchers Supporting Project number (PNURSP2023R161), Princess Nourah bint Abdulrahman University, Riyadh, Saudi Arabia. This study is supported via funding from Prince Sattam bin Abdulaziz University project number (PSAU/2023/R/1444). The authors would like to thank the Deanship of Scientific Research at Umm Al-Qura University for supporting this work by Grant Code: (22UQU4310373DSR62). The Authors would like to thank the Deanship of Scientific Research at Majmaah University for supporting this work under Project No. R-2023-68.”

The original Article has been corrected.

